# Tailless and filamentous prophages are predominant in marine *Vibrio*

**DOI:** 10.1093/ismejo/wrae202

**Published:** 2024-10-18

**Authors:** Kerrin Steensen, Joana Séneca, Nina Bartlau, Xiaoqian A Yu, Fatima A Hussain, Martin F Polz

**Affiliations:** Centre for Microbiology and Environmental Systems Science, Division of Microbial Ecology, University of Vienna, Djerassiplatz 1, 1030 Vienna, Vienna, Austria; Doctoral School in Microbiology and Environmental Science, University of Vienna, Djerassiplatz 1, 1030 Vienna, Vienna, Austria; Centre for Microbiology and Environmental Systems Science, University of Vienna, Djerassiplatz 1, 1030 Vienna, Vienna, Austria; Joint Microbiome Facility of the Medical University of Vienna and the University of Vienna, Djerassiplatz 1, 1030 Vienna, Vienna, Austria; Centre for Microbiology and Environmental Systems Science, Division of Microbial Ecology, University of Vienna, Djerassiplatz 1, 1030 Vienna, Vienna, Austria; Centre for Microbiology and Environmental Systems Science, Division of Microbial Ecology, University of Vienna, Djerassiplatz 1, 1030 Vienna, Vienna, Austria; Department of Civil and Environmental Engineering, Massachusetts Institute of Technology, 15 Vassar St., Cambridge MA 02138, United States; Centre for Microbiology and Environmental Systems Science, Division of Microbial Ecology, University of Vienna, Djerassiplatz 1, 1030 Vienna, Vienna, Austria

**Keywords:** prophage, tailless phage, filamentous phage, Vibrio, induction, integration, phage defense

## Abstract

Although tailed bacteriophages (phages) of the class *Caudoviricetes* are thought to constitute the most abundant and ecologically relevant group of phages that can integrate their genome into the host chromosome, it is becoming increasingly clear that other prophages are widespread. Here, we show that prophages derived from filamentous and tailless phages with genome sizes below 16 kb make up the majority of prophages in marine bacteria of the genus *Vibrio*. To estimate prophage prevalence unaffected by database biases, we combined comparative genomics and chemical induction of 58 diverse *Vibrio cyclitrophicus* isolates, resulting in 107 well-curated prophages. Complemented with computationally predicted prophages, we obtained 1158 prophages from 931 naturally co-existing strains of the family *Vibrionaceae*. Prophages resembling tailless and filamentous phages predominated, accounting for 80% of all prophages in *V. cyclitrophicus* and 60% across the *Vibrionaceae*. In our experimental model, prophages of all three viral realms actively replicated upon induction indicating their ability to transfer to new hosts. Indeed, prophages were rapidly gained and lost, as suggested by variable prophage content between closely related *V. cyclitrophicus*. Prophages related to filamentous and tailless phages were integrated into only three genomic locations and restored the function of their integration site. Despite their small size, they contained highly diverse accessory genes that may contribute to host fitness, such as phage defense systems. We propose that, like their well-studied tailed equivalent, tailless and filamentous temperate phages are active and highly abundant drivers of host ecology and evolution in marine *Vibrio*, which have been largely overlooked.

## Introduction

When comparing genomes of closely related bacteria, a frequently encountered difference is variation in their integrated bacteriophage genomes (prophages). These viruses, also called temperate phages, can be vertically inherited while genomically inserted in their host’s chromosomes or transferred horizontally by prophage activation (induction) and subsequent infection of new hosts. Prophages are widespread bacterial parasites that commonly lead to host lysis upon induction. They also impact their host’s phenotype by carrying accessory genes, such as those involved in antibiotic resistance [1], toxin production [2], and bacteriophage (phage) defense [3–7]. This diversity of additional functions seems to represent a source of novel genetic material for bacterial hosts in natural environments, as temperate phages are rapidly gained and lost [8–10]. However, most of our knowledge on prophage diversity is based on studies using prediction tools for the identification of genomically inserted prophages [11–14]. These tools heavily rely on known prophages deposited in public databases, which are biased towards tailed dsDNA phages of the class *Caudoviricetes* [15]. Tailed phages are characterized by the classical head and tail structure, using HK97-fold proteins for their icosahedral capsids. Even though most phage biologists are aware of the large bias towards tailed phages, only a few studies have focused on other prophage types. Several computational studies specifically targeting prophages derived from filamentous and tailless phages suggest that these prophages are more widespread than previously thought [16–19].

Filamentous phages of the order *Tubulavirales* differ from the tailed *Caudoviricetes* both morphologically as well as in their mode of infection and reproduction. Unlike most other prophages, they are secreted without killing their host. Instead, their 5–15 kb small, single-stranded DNA genomes are packaged into filamentous virions that are extruded through the bacterial membrane [20]. Although specific filamentous phages, such as the phage CTXφ involved in the virulence of *Vibrio cholerae*, have been intensively studied in the context of disease for decades, the large scope of their natural diversity has only been recently uncovered [2, 17].

Prophages belonging to the order *Vinavirales* are even less well studied but have been reported to occur in a diversity of bacterial species and different environments, such as in seawater or the human gut [16, 18, 19]. These prophages that are commonly related to the tailless corticovirus PM2 are inducible spontaneously or after Mitomycin C (MMC) treatment [19, 21, 22]. In contrast to tailed phages, tailless phages possess an icosahedral capsid formed by double jelly-roll-fold major capsid proteins, which contains a lipid layer covering their dsDNA genome. These characteristics*,* often unaccounted for in standard phage isolation protocols, might introduce an isolation bias further impeding their discovery as shown for other tailless viruses before [21].

Although it is becoming increasingly evident that prophages belonging to the *Vinavirales* and *Tubulavirales* are more widespread than previously thought, their abundance has not been evaluated in the context of other prophages, and how they impact their hosts is largely unexplored. Therefore, we aimed at obtaining a holistic view of the prevalence and diversity of prophages in a coastal ocean isolate collection of *Vibrio*, a bacterial genus that has been reported to carry prophages belonging to the *Vinavirales*, *Tubulavirales*, and *Caudoviricetes*, that we will refer to as “tailless”, “filamentous”, and “tailed” prophages for better readability in this manuscript [19, 21, 23, 24]. We show that filamentous and tailless prophages are more abundant (~80%) than tailed prophages in a well-curated model system of 58 *Vibrio cyclitrophicus* strains using a combination of comparative genomics and chemical induction. This trend was confirmed in over 900 strains of naturally co-occurring marine *Vibrionaceae*. Although most detected prophages had small genomes below 16 kb, they often harbored accessory genes. Prophages were frequently gained and lost, as highlighted by their high diversification among closely related hosts. Our comprehensive prophage collection illustrates that tailless and filamentous prophages have been systematically underestimated in abundance and suggests that they are important drivers of host diversification in environmental bacteria.

## Material and methods

### Bacterial isolation and growth conditions

Bacteria were isolated in previous studies from coastal seawater collected in two close-by sampling locations namely Plum Island Sound Estuary, Ipswich, MA in 2007 and Canoe Grove, Nahant, MA in 2010 as described before [25–28]. In brief, *Vibrio* were isolated by applying a size fractionation approach followed by cultivation on TCBS selective media. If not otherwise stated, *Vibrio* isolates were cultivated using 2216 Marine Broth “2216 MB” (Difco) at 25°C.

### *Vibrionaceae* genome collection

For our experimental model system, we sequenced high-quality genomes of 58 *V. cyclitrophicus* strains in several rounds of third generation long-read sequencing (Oxford Nanopore) from bacterial cell pellets (Supplementary Methods). For prophage prediction across different species from the family *Vibrionaceae*, we used publicly available genomes, complemented with newly generated genomes (Table S2). The newly generated *Vibrionaceae* genomes were either sequenced as described previously [21], or sequenced as follows: Genome libraries were prepared using the Nextera DNA Flex library preparation kit (Illumina) utilizing a liquid handler, and then sequenced at 2x150 bp on a Nextseq 550 platform (Illumina). Reads were preprocessed by removing adaptors using hts_SuperDeduper with parameters “*start = 5, min_length = 40*” (v1.1.0, https://github.com/s4hts/HTStream) and phiX sequences were removed using bowtie2 (v2.3.4.2, (Langmead and Salzberg, 2012)). Reads were then quality-trimmed using Trim Galore with the specifications “*length = 30, stringency = 1, error_rate = 0.05, max_n = 1*” (v0.5.0, https://github.com/FelixKrueger/TrimGalore) and downsampled using a digital normalization approach implemented in the normalize-by-median.py script using the parameters “*kmer_size = 22, coverage_cutoff = 20, fp_rate = 0.8*” from the kmher software package (v3.0.0a1, [29]). The remaining reads were assembled using spades (v3.12.0, [30]) with the flag “—*careful*”.

### Phylogeny of closely related *vibrio cyclitrophicus*

The phylogenetic relationship of *V. cyclitrophicus* hosts was calculated as follows: First, we aligned the host core genome with Mugsy (v1.2.3, [31]), and used the alignment to construct a maximum-likelihood tree using PhyML (v3.3, [32]) with a General Time Reversible substitution model, as well as empirical models for the equilibrium nucleotide frequency, nucleotide substitution rates, and the gamma distribution shape parameter (*−m 'GTR' -t 'e' -a 'e' -f 'm' -v 'e'*). We then inferred the vertically inherited and recombined regions in the core genome alignment using ClonalFrameML (v1.12, [33]), with the maximum-likelihood tree and the transversion to transition rate kappa estimated during tree construction as input, and 100 bootstrap replications (*−emsim 100)*. Recombinant regions were masked using maskrc-svg (https://github.com/kwongj/maskrc-svg), and the masked alignment was used to calculate a new maximum-likelihood tree using the same settings in PhyML.

### Prophage identification in *Vibrio cyclitrophicus*

#### Prophage candidate identification using comparative genomics

To get an unbiased prediction of prophages in the 58 *V. cyclitrophicus* strains, we used a graph-based approach and identified 1286 flexible genome elements using PPanGGOLiN in the panrgp mode (v1.2.105, [34]).

#### Sequencing of MMC induced cultures

To identify additional active prophages and distinguish prophages from non-phage mobile genetic elements, we treated bacterial cultures with MMC to induce prophages and subsequently sequenced the whole culture. In detail, *V. cyclitrophicus* strains (n = 58) were incubated on 1.5% 2216 MB streak plates overnight. Single colonies were transferred into 5 ml liquid 2216 MB and allowed to grow overnight shaking at 250 rpm. Then, cultures were diluted 1:100 into 4 ml fresh medium in glass tubes, grown to an OD_600nm_ ~ 0.2, and then MMC (Sigma-Aldrich, M0503) was added to a final concentration of 0.5 μg/ml. After MMC addition, 200 μl culture were transferred to a 96-well plate. OD_600nm_ was measured every 5 min shaking at 240 rpm with the Tecan Spark plate reader. Culture tubes were shaken at 250 rpm in the dark for either 6 h or 14 h depending on if the optical density dropped before 6 h (= sampling after 6 h) or not (= sampling after 14 h).

We extracted DNA of 500 μl induced bacterial culture using the Blood and Tissue kit (QIAGEN) according to the manufacturer’s protocol with adjustments: The cell lysis step using Proteinase K and lysis buffer (QIAGEN) was increased to 15 min to ensure capsid disruption. DNA was eluted in 100 μl AE buffer (QIAGEN) and quantified using the Qubit dsDNA HS kit (Invitrogen). Barcoded sequencing libraries were prepared for sequencing using the NEBNext Ultra II DNA Library Prep Kit for Illumina (New England Biolabs) and NEBNext Multiplex Oligos for Illumina (New England Biolabs) and sequenced on a MiSeq using a v3 kit in 2x 300 bp mode (illumina).

#### Identification of active prophages

To identify the genomic location of the excised prophage elements, we mapped reads of induced cultures against the high-quality reference genomes. Then, we identified regions with increased read coverage compared to the host’s background coverage adapted from Zünd et al. [35]. We removed sequencing adaptors and phiX residues from the reads, that were then quality filtered (*qtrim = r trimq = 14 minlen = 45 maq = 20 ftl = 12)* using BBduk from the BBMap-suite (v. 39.01, [36]). The trimmed reads were mapped against their respective host reference genome using the burrows-wheeler local aligner bwa-mem (v0.7.17, [37]). We filtered the mapping output to obtain only the primary alignment to avoid reads mapping to more than one location, such as repeats, using samtools (v1.19) view with the flags *-h -F 260* [38]. The alignments were filtered for an identity > = 99%, alignment length of > = 50 bp, and a read coverage of at least 80% using code adapted from mVIRs [35]. The filtered file was sorted by name and a coverage “depth” profile was created using samtools [38]. We proceeded with samples >250 000 reads resulting in a mean read coverage ranging from 6 to 95.

To identify high coverage regions, we used a custom python script. We set a tailored median *neighborhood* coverage and corrected standard deviation for each contig longer than 80 kb, instead of using values for the complete genome. To avoid natural skew of the coverage along very long contigs, we sliced contigs >350 kb into pieces of equal length and no more than 300 kb. The corrected standard deviation was calculated using the standard deviation excluding all extreme coverage values >(3 ^*^ median) to avoid that actively excising elements influence the corrected standard deviation of the background. The coverage along the host reference genome was then compared to the tailored median coverage value using a sliding window approach. To account for possible prophage plasmids, contigs between 5–80 kb with an elevated coverage of more than one standard deviation above the genome median coverage (median_contig_ > median_genome_ + 1^*^ std_contig_) were completely recorded as high coverage. To identify elements with elevated coverage across the chromosome, we calculated the mean coverage of a sliding window of 250 bp and a step-size of 50 bp. Regions were considered as high coverage if consecutive windows resulted in elements that are 500–1500 bp long with coverage >(median + 3^*^standard deviation) or > 1500 bp with a coverage >(median + 1^*^standard deviation). Overlapping elements, or elements being less than 1000 bp apart from each other were combined, including those that spanned contig borders. We manually inspected all genome coverage plots adding slightly elevated elements not found by the algorithm and expanding or decreasing the size of elements based on the base coverage values if necessary.

#### Prophage candidate curation by gene content

We combined all elements identified by induction and comparative genomics and eliminated non-phage elements as follows: We predicted genes using prodigal in the meta mode (v2.6.3, [39]). and annotated them using hmmsearch (hmmer v3.4, [40]) against VOGdb (v221, [41]) using an e-value of 0.001. Elements were considered as putative prophages if they contained at least five genes and a) either contained a clear marker gene, that were terminase (large/endonuclease subunit) or major capsid proteins for tailed phages, a pI-like ATPase for filamentous phages or a double jelly-roll capsid protein for tailless phages or b) contained at least two genes indicating a viral origin and contained the words 'coat', 'capsid', 'head', 'tail', 'virion', 'phage', 'holin', 'terminase', 'replication', 'packaging', as well as filamentous phage associated keywords 'Vpf81' and pI-like ATPase hits namely 'BPPHL_Gene_1_protein' and 'BPFD_Gene_1_protein'. The resulting prophage candidates were merged and manually inspected to distinguish between prophages and other microbial genetic elements. Finally, we ensured prophage candidates to be single, distinct elements by manually inspecting gene predictions and alignments of all identified prophage candidates using clinker (v0.0.28, [42]) Adjacent prophages were separated. Additional elements next to the phage that were absent in most other related prophages were discarded. The resulting 107 elements, of which 106 were identified by comparative genomics and 65 by chemical induction, were considered to be prophages.

#### Prophage border curation

We predicted the prophage borders accurately by combining the results obtained from comparative genomics and the induced prophage coverage data. We used a hierarchical approach: First, we checked for a sufficient number of “clipped” reads close to the predicted borders calculated by mVIRs with the flags -ml 1000 -ML 100000 -m (v1.1.1–3.9.16, [35]). These reads are spanning the part of a circularized phage that is connected to the host genome upon integration. While one part of the read maps well to the host reference, the other part of the read gets clipped at the transition from host to the integrated prophage and therefore provide exact positions of the prophage borders. Second, if clipped reads were unavailable but the phage was actively induced, we used the base coverage of the prophage region to determine the prophage borders. Otherwise, we used the borders predicted by PPanGGOLiN covering the prophage from its first to its last gene discarding the non-coding ends. For phages inserted in a plasmid, the border was set to the first gene not shared with other plasmids having a similar backbone.

### Prophage identification across the *Vibrionaceae*

#### Marker gene collection

We created a marker gene collection from the predicted prophages in *V. cyclitrophicus.* We obtained these genes by creating an all-against-all protein similarity matrix using blastp with an e-value of 0.00001 and subjected it to the markov clustering tool mcl (v22.282) (mcxload with options *--stream-mirror --stream-neg-log10 -stream-tf 'ceil(200)'*) using an inflation factor of 3. Clusters containing marker genes, that were terminase (large/endonuclease subunit) and major capsid proteins for tailed prophages, pI-like ATPase proteins for filamentous prophages and double jelly-roll major capsid proteins for tailless phages were merged into a single marker gene collection per viral realm.

#### Predicting related phages across the Vibrionaceae and in *V. Cyclitrophicus* draft genomes

We ran ppanngolin (v1.2.105 [34],) species-wise in *panrgp* mode to obtain genomic islands of all strains with clear taxonomic assignment containing at least five members. First, we used blastp (v2.15.0 [43],) to compare genes from our custom-made marker gene collection for each viral realm against the genomic islands. Second, we ran the viral gene search described in “Prophage candidate curation by gene content” and sorted elements into the respective viral realms or “other”. We applied a viral-realm specific size filtering step (filamentous/other 4 kb, tailless 10 kb, tailed 25 kb). Prophages that were largely disrupted or not showing integrity with related prophages were excluded.

To investigate how the use of fragmented genomes influences prophage identification, we repeated the genomic island prediction and viral gene search in *V. cyclitrophicus* using genomes assembled from next generation sequencing data (Table S2) with an average contig count of 180.6 compared to 3.8 in high quality genomes. We then checked if they were found in both high-quality and draft genomes of *V. cyclitrophicus* using blastn (v2.15.0, [43]).

### Prophage annotation and classification

We annotated the curated prophages with accurate borders from *V. cyclitrophicus* (Table S3) and all prophages across the *Vibrionaceae* with a custom pipeline: Genes were predicted using prodigal in the meta mode (v2.6.3, [39]). Genes were annotated using *hmmsearch* (hmmer v3.4, [40]) against VOGdb (v221) using an e-value of 0.0001, hmmscan (evalue 0.001) against the antidefense database APIS (2023–09) [44], Defensefinder (v1.2.2 [45, 46], blastp (v2.15.0, [43]) against the Swiss-Prot database (last access in 03–2024, [47]), and interproscan (v5.67–99.0-11.0.4, [48]) using the applications TIGRFAM, SUPERFAMILY, ProSiteProfiles, and Pfam.

Viral realms were determined as follows: Prophages containing a double jelly-roll capsid were grouped into the realm *Varidnaviria* (“tailless phages”). Prophages containing a pI-like ATPase, predicted as “gene 1” or “zonular occludens toxin”, were grouped into the *Monodnaviria* (“filamentous phages”) and prophages containing capsid or a terminase (large/endonuclease subunit) were grouped into the realm *Duplodnaviria* (“tailed phages”). The viral groups were further classified using the VICTOR web service [49] by pairwise comparison on nucleotide level using the Genome-BLAST Distance Phylogeny (GBDP) method [50] and subsequent clustering into genera using the recommended clustering thresholds and an F value of 0.5. The resulting phylogenomic GBDP trees were inferred with FASTME [51] using the formula D0 and yielded an average support of 49% (tailless), 58% (filamentous), and 59% (tailed). we considered VICTOR’s GBDP method to be better suited for this analysis, because similarity-based approaches as used in VIRIDIC [52] would be highly influenced by the variable locus causing very small clusters.

### Comparison to known prophage sequences

We phylogenetically classified the identified prophages per viral realm by running blastp (bit score > 50, e-value <0.00001, (v2.15.0) [43] of their predicted proteins against the INPHARED database (March 2024, [53]). We considered results with genome sizes of 50%–150% of the query phage genome and sharing > = 5% of query phage genes. We calculated a tree of all hits combined with the identified prophages using VipTree (v1.1.2) [54] and extracted sequences clustering together. We then calculated a phylogenetic tree with the identified prophages together with selected representatives of known viruses (Table S4) based on the INPHARED search and current ICTV classification (International Committee on Taxonomy of Viruses (ICTV): https://ictv.global/taxonomy/) using VICTOR [49] as described above. In addition to the whole-genome trees, we also calculated trees of the respective marker genes for each group using mafft (v.7.525, [55]) and fasttree (v.2.1.11, [56]) with standard settings. We checked whether current prophage prediction tools recognized the 107 well-curated prophages as viruses using VirSorter2 (v.2.2.4, [15]) with standard settings and geNomad (v.1.8.0, [57]) in end-to-end mode.

### Structural analysis of double jelly-roll major capsid proteins

We extracted the amino acid sequence of the genes predicted as double jelly-roll major capsid proteins of reference phage NO16 and selected tailless prophages. Structural predictions were performed using the AlphaFold Server powered by the AlphaFold 3 model [58] and visualized using chimeraX [59]. The crystal structure of the major capsid protein of PM2 [60, 61] was downloaded from the PDB database.

### Prophage activity analysis

We used sequenced MMC induced cultures to estimate prophage activity. We first calculated the mean sequencing coverage of each curated prophage element and compared it to their tailored median neighborhood coverage and corrected standard deviation for that specific region described in “Identification of active prophages”. Prophages were deemed active if their mean coverage exceeded the median coverage of their genomic neighborhood by > = one corrected standard deviation, or highly active if the median coverage was exceeded by > = three corrected standard deviations. We defined the phage-to-host ratio (PtoH) by dividing the prophage mean coverage by the median neighborhood coverage and further used it as a proxy for phage activity. Plasmid-associated phage activity and PtoH values were refined by first comparing the mean coverage against the whole genome median and multiplied by a plasmid correction factor retrieved from the contig coverage of the original non-induced nanopore reads against the reference genome. We only applied the correction factor if plasmids were present in multiple copies meaning that the corrected PtoH could only decrease.

We checked if tailed and tailless phage activity significantly differed by comparing their PtoH values. Thus, we applied the Shapiro–Wilk test, which showed a non-normal distribution, followed by the Wilcoxon rank sum exact test.

### Prophage transfer analysis

#### Prophage similarity analysis

To identify recently transferred prophages, we calculated the similarity of all identified prophages in *Vibrio* as average nucleotide identity using fastANI (v.1.34, [62]). We then filtered all prophage pairs for an identity of at least 99.9% and complete coverage.

#### Host *phylogeny*

To classify *Vibrionaceae* genomes on species level (Table S2), a phylogenetic tree of the concatenated ribosomal proteins was calculated using RiboTree (https://github.com/philarevalo/RiboTree) using the *Vibrionaceae* RefSeq genomes (downloaded November 2023 from NCBI) as reference. In brief, we removed all contigs <1000 bp from the genomes and ran RiboTree with 100 bootstraps using *Shewanella denitrificans* (GCF_000013765.1) as outgroup. Genomes were classified accordingly if they clustered with the RefSeq genomes. Strains not clustering into species were excluded from the following analysis.

The *V. cyclitrophicus* tree used for inference of the prophage transmission type was calculated as described in “Phylogeny of closely related *V. cyclitrophicus*”.

### Variable locus analysis

To find accessory gene loci in filamentous and tailless prophages identified in the *Vibrionaceae*, we built fine-grained prophage clusters based (VGCs) using VirClust with standard settings and a clustering distance of 0.6 [63]. We dereplicated the prophages at 99.9% using cd-hit resulting in 317 (out of 401) tailless and 239 (out of 302) filamentous prophages (v4.8,1, [64]). Prophage-encoded proteins were assigned to gene families by mcl clustering on a blastp generated protein similarity matrix (*e-value 0.00001*) with the settings ‘*—stream-mirror —stream-neg-log10 -stream-tf 'ceil(200)"* [43, 65] and we calculated their frequency within the prophage clusters. Gene families flanking the variable loci were identified by manual inspection of the synteny plots. We excluded clusters with <5 representatives or not displaying variable genes enclosed by two core phage genes (present in >80% of the phages). We recorded each variable locus from the first to the last variable gene (present in <=30% of the phages) between the respective core genes resulting in 118 filamentous and 243 tailless protein-encoding variable loci and annotated the genes with our custom pipeline (comp. “Prophage annotation and classification”). Defensive genes were reported if found by Defensefinder, or if their annotation was related to abortive infection or toxin/antitoxin systems. Systems were considered complete when reported by Defensefinder, or if neighboring toxin and antitoxin genes were found.

### Integration site analysis

#### Location and functional annotation of integration sites

Prophages integration sites, containing at least one prophage in *V. cyclitrophicus* (Table S1), were inferred using PPanGGOLiN (v1.2.105, [34]). We blasted the flanking regions of selected prophages per hotspot against the genome of 10 N.286.51.B1, a *V. cyclitrophicus* strain with no occupied prophage hotspots, to locate the integration sites. To confirm that filamentous phages were integrated at the *dif* sites, we used blastn tailored for short sequences *(−task blastn-short -word_size 12*) (v2.15.0, [43]) to compare the integration site and the prophages themselves against previously published *dif* sequences from *V. cholerae* [66]. To check for the functional annotation of hotspot 1, we computationally excised the tailless prophage integrated in hotspot 1 at its predicted borders (comp. Prophage border curation) and inspected the stitched region for their functional annotation assigned by the NCBI Prokaryotic Genome Annotation Pipeline [67].

#### Integrase trees

We calculated trees of the integrase genes of both tailless and tailed phages running mafft (v.7.525, [55]) and subsequently fasttree (v.2.1.11, [56]) with standard settings. Filamentous phages were excluded because they do not regularly encode integrase genes.

### Data analysis, tools and visualization

All analyses were perfomed using bash and python (v3.12.1). We used the libraries pandas (v2.1.4), matplotlib (v3.8.2), numpy (v1.26.3), and seaborn (v0.12.2). Statistical analyses were conducted in R (v4.3.2). We used ChatGPT (GPT-4) to improve grammar and clarity of the text. Trees were visualized using iToL [68] or ggtree (v3.8.2 [69],). The heatmap was plotted in R (v4.3.2) using the additional packages phangorn (v2.11.1 [70],), ggplot2 (v3.4.4 [71],) with helper functions from reshape2 (v1.4.4), patchwork (v1.1.3), dplyr (v1.1.4), and RColorBrewer (v1.1–3). Figures were edited with Inkscape (v1.1.2).

## Results

### *V. cyclitrophicus* harbor prophages from three viral realms

**Figure 1:**
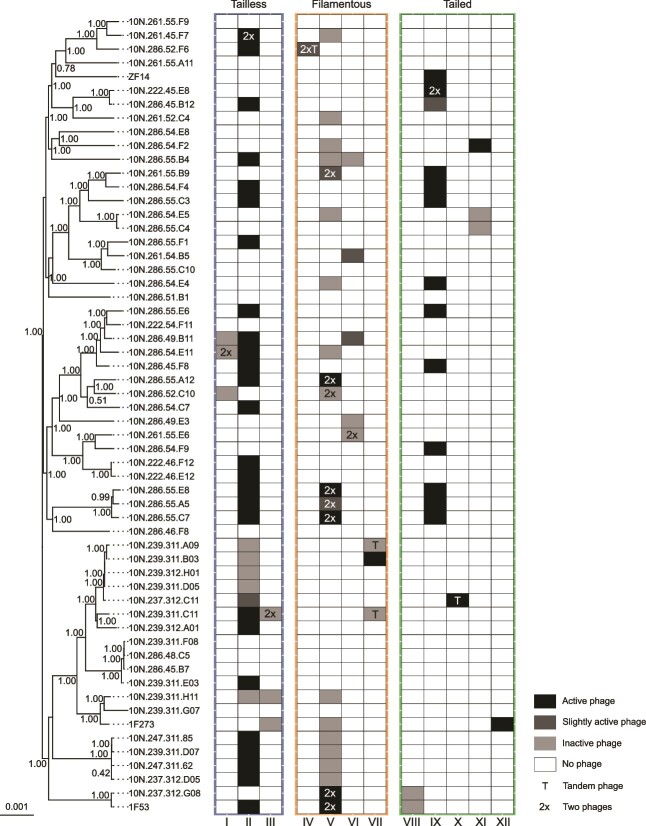
Prophage presence in a set of 58 *V. Cyclitrophicus* isolates. Clustermap showing the recombination-free phylogenetic tree of 58 *V. Cyclitrophicus* along with the presence and activity of induced prophages in the respective bacterial strains. Bootstrap values ranging from 0 to 1 are indicated at the nodes, and the tree scale shows the nucleotide substitutions in the core genome comprising 3 785 366 bp. Columns I-XII reflect VICTOR-predicted genera of prophages belonging to the three different viral realms. Temperate phage activity measured as coverage increase over the host genome in MMC-induced cultures is depicted in greyscale (active: > median + 3^*^std, slightly active: > median coverage +1^*^std, inactive: < median coverage +1^*^std). The labels inside the heatmap denote two similar prophages co-occurring in the same integration site (T) or in different locations in the genome (2x).

Using a combination of comparative genomics and chemical induction, we identified 107 prophages in 58 marine *V. cyclitrophicus* strains, which were isolated from the same coastal area (Fig. 1, Table S1-S2). First, we predicted genomic islands from high-quality *V. cyclitrophicus* genomes, exploiting the fact that prophages belong to the flexible genome. To better differentiate prophages from other mobile genetic elements, we then sequenced DNA from cultures that were exposed to mitomycin C (MMC), which can induce prophages via the host’s SOS system. All genomic islands and induced elements were combined, filtered based on their viral gene content (Table S3) and manually curated. This pipeline resulted in a prophage dataset with accurately predicted prophage boundaries (methods), in contrast to prophage prediction tools, which oftentimes imprecisely predict prophage borders [35]. Our comprehensive approach revealed that prophages are common with an average prophage count of 1.8 and > 80% of the investigated strains carrying at least one prophage (Fig. S1), which is in accordance with previously estimated prevalence among diverse bacteria [72].

We found a large diversity of prophages spanning three distinct viral realms: tailless prophages of the order *Vinavirales* possessing a double jelly-roll-fold major capsid protein characteristic for viruses of the realm *Varidnaviria*, filamentous prophages containing the pI-like ATPase marker gene and belonging to the order *Tubulavirales* assigned to the realm *Monodnaviria* and tailed prophages containing a HK97-fold capsid typical for orders within the *Caudoviricetes* from the realm *Duplodnaviria* [17, 73]. Even though filamentous and tailed prophages have been reported together [12], we show that prophages from all three realms can co-occur in single strains of *V. cyclitrophicus* (Fig. 1). Classifying each prophage group using a nucleotide sequence-based approach [49] resulted in 12 genera (I-XII, Fig. 1). We compared prophage genomes and their marker genes from all genera to publicly available viral genomes (Fig. S2 and S3, Table S4) and, additionally, checked whether the prophages are recognized by two current prophage prediction tools, VirSorter2 [15] and geNomad [57] (methods).

**Figure 2:**
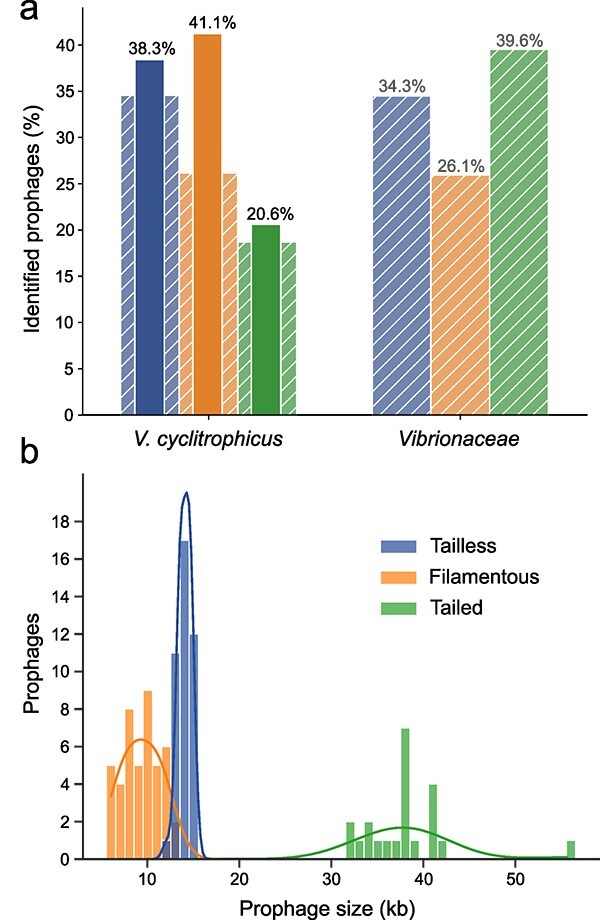
Prophage distribution and genome size in the *Vibrionaceae*. a) Proportion of each viral realm in the total number of prophages identified in 58 *V. Cyclitrophicus* strains and in a broader set of 931 bacterial strains across the *Vibrionaceae* including the 58 *V. Cyclitrophicus* strains. Fully colored data indicate highly curated prophages predicted in high-quality genomes. Hashed data indicate computational prediction in draft genomes. b) Histogram showing the genome size of prophages in *V. Cyclitrophicus* colored by their respective viral realm.

Genomes from the three tailless prophage genera did not cluster with any other tailless phages on genus level (Fig. S2a). Despite low amino acid identity, the identified prophages shared blocks of similar open reading frames with the phages PM2 and NO16 (Fig. S4a) suggesting that the identified prophages also belong to the *Corticoviridae*. Such PM2-like prophages have previously been found in diverse marine bacteria [16, 19]. Comparisons of the double jelly-roll major capsid protein, however, show that all three genera cluster together next to capsid proteins originating from *Autolykiviridae* viruses sampled in the same coastal area (Fig. S3a). The high nucleotide identity among double jelly-roll major capsid proteins of all different tailless phages observed in our *Vibrio* collection might be a result of their shared environment including similar bacterial hosts. This raises the question whether genome structure might be more meaningful for the classification of tailless phages, as conserved synteny has been noticed in tailless phages before [74]. Most identified tailless prophages from genera I and II were recognized as viral by at least one prophage prediction tool. However, prophages of genus III were never detected (Table S1). These appeared more distantly related to other tailless phages (Fig. 2a) and were exclusively encoded on plasmids carrying various mobile genetic elements (Fig. S4b). Given that the structural prediction of their double jelly-roll major capsid proteins resembled those of capsid proteins encoded by other tailless phages (Fig. S5), genus III might represent a distinct group of tailless phages.

In contrast to the tailless prophages, most filamentous and tailed prophages were related to viruses from known genera (Fig. S2b-c) and each identified prophage was also recognized by at least one prophage prediction tool (Table S1). Whole genome comparisons suggested that prophages from the most prevalent filamentous prophages belonging to genus V were related to fibrovirus fs1, which has been reported to release large amounts of phage particles into the supernatant of host cultures [75]. Consistently, several filamentous phages from genus V appeared active upon MMC induction as indicated by an increase in the coverage compared to their host’s genomes (Fig. 1). However, their pI-like ATPase genes seemed more closely related to those of multiple other inoviridae genera (Fig. S3b). Filamentous prophages of genus VII did not appear to be closely related to known viruses, but occasionally occurred as tandemly integrated elements as known from other filamentous phages [17, 76]. Most tailed prophages from our dataset belonged to genus IX and resembled myoviruses of the family *Peduoviridae* (Fig. S2c), similar to lytic tailed phages previously isolated from the same coastal area [21].

### Filamentous and tailless prophages are highly prevalent across the *Vibrionaceae.*

Both tailless and filamentous prophages outnumbered tailed prophages in our model system *V. cyclitrophicus*. Filamentous prophages were the most prevalent group (40.4% of total), followed by tailless prophages (37.6% of total) compared to relatively few detected tailed prophages (22% of total) (Fig. 2a). The predominant filamentous and tailless prophages had small genome sizes, ranging between 6.0–13.3 kb for filamentous phage genomes and 12.1–15.1 kb for tailless phage genomes. Tailed prophages were larger ranging from 32.4 to 42.1 kb and one prophage with 55.6 kb exceeded this size range (Fig. 2b). With the predominant prophages having genomes smaller than 16 kb, the size distribution we report here is in contrast to studies reporting the average genome size of prophages to be above 30 kb [72, 77].

To investigate whether this prophage distribution was limited to *V. cyclitrophicus*, we expanded our search to elements related to the identified prophages in a broader set of genomes of 11 species belonging to the family *Vibrionaceae* that co-exist with *V. cyclitrophicus* in the same coastal environment (Tables S1–S2). We observed that tailed prophages were most abundant (39.6%), followed by tailless (34.3%), and filamentous (26.1%) prophages (Fig. 2a). Whereas the entire set of *V. cyclitrophicus* genomes were closed or nearly so, prophage identification across the expanded dataset of *Vibrionaceae* may be influenced by their incomplete assembly. Therefore, we additionally predicted prophages in draft genomes of the 58 *V. cyclitrophicus*, which indeed suggests a systematic underestimation of filamentous prophages (Fig. 2a), of which we could identify only 28 in the draft genome set in comparison to 44 in high-quality genomes. Therefore, the observed abundances suggest that all three viral realms are roughly evenly represented in the totality of prophages in the *Vibrionaceae*.

### Prophages of all viral realms appear to actively replicate

We aimed at measuring prophage activity by sequencing MMC-treated bacterial cultures after 6 or 14 h of growth (Table S2) and subsequently mapping the reads against a host reference genome. The calculated phage-to-host ratio (PtoH) serves as a proxy for the number of phage particles being produced per cell (methods). Although this assay also includes spontaneously induced prophages, the majority of prophages appeared inducible by MMC, a known activator of the SOS response in bacteria [78].

We found that prophages from all three viral realms amplified their genomes upon MMC treatment (Fig. 1). About 48% demonstrated high activity (coverage >median + 3^*^std) 11% seemed to replicate at rather low levels (coverage >median + 1^*^std) and 41% of the prophages identified in *V.cyclitrophicus* appeared to be inactive. To better understand what influences the number of produced phage particles, we checked if the number of produced phage particles might be predicted by their viral realm. Even though the tailless phage average PtoH ratio of 135 was higher than 79 in tailed phages (Fig. S6), we did not find evidence for a significant difference in tailed and tailless phage activity (Wilcoxon rank sum test, *P* = 0.6419). We additionally observed that three identical tailless phages had highly variable PtoH ratios under similar experimental conditions in different strains of *V. cyclitrophicus*. Therefore, the number of produced phage particles could also be linked to host features such as growth or its genome architecture including inserted microbial genetic elements as additionally inserted prophages have been shown to change the number of formed phage particles upon induction [79, 80].

Filamentous phages generally demonstrated lower phage-to-host ratios than other phages, probably caused by their ssDNA genome not being captured by the sequencing technique. The slightly increased PtoH values likely reflect the double stranded replicative form of filamentous phages occurring during replication [81, 82]. Therefore, we did not compare filamentous phage activity against the PtoH values of tailless and tailed phages.

**Figure 3:**
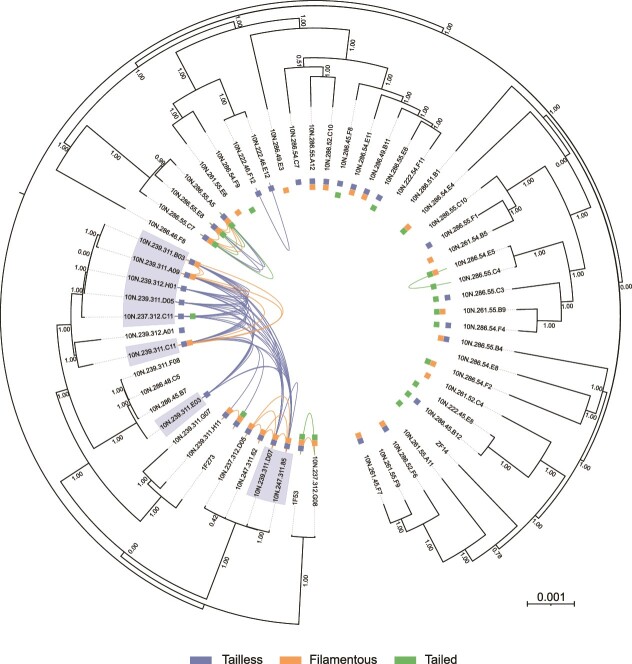
Prophage transfer and inheritance within 58 co-occurring *V. Cyclitrophicus*. Similar prophages (ANI > = 99.9%, full coverage) shared between different strains of *V. Cyclitrophicus* are depicted as connections by viral realm. The tree shows the relationship of bacterial hosts calculated using a recombination-free alignment of the host core genome. Bootstrap values ranging from 0 to 1 are indicated at the nodes, and the tree scale denotes nucleotide substitutions in the core genome comprising 3 785 366 bp. Hosts carrying prophages are marked with boxes according the associated viral realm. Boxes without any connections indicate that the prophages were unique, i.e. not highly similar to other prophages. Hosts denoted by boxes carry a similar tailless prophage that seems to currently expand in the population by horizontal gene transfer between, and vertical inheritance within branches.

### Prophages are rapidly gained and lost

Having identified prophages from multiple *Vibrio* species co-existing in the same environment, we aimed at estimating the dynamics of prophage gain and loss in the context of host diversity. To constrain these dynamics to more recent timescales, we only considered closely related prophages that recently descended from the same ancestor. We therefore carried out an all-against-all comparison of the 1158 prophages found across the *Vibrionaceae* and selected prophages with an average nucleotide identity ≥99.9% and full sequence coverage to allow for single nucleotide substitutions (methods). We observed that the 1474 pairs of highly similar prophages we retrieved were primarily found in hosts of the same species (Fig. S7) suggesting that in their recent history, horizontal transfer of temperate phages between different *Vibrio* species was rare. To further explore the dynamics within a species, we focused on our well-curated model system *V. cyclitrophicus*. To infer the evolutionary distance between hosts, we computed a phylogenetic tree unaffected by changes in the flexible genome and homologous recombination (methods). Interestingly, highly similar prophages were still limited to more closely related *V. cyclitrophicus* strains, indicating that temperate phages are specific to hosts on the sub-species level (Fig. 3).

We observed prophages from all three viral realms to be rapidly gained and lost within *V. cyclitrophicus*. This was demonstrated by variable prophage content even among the most closely related sisters on the tree (Fig. 3), which has been associated with rapid mobile genetic element turnover before (e.g. 9,10). Strikingly, most identified prophages were unique (n = 62/107) as they were not highly similar to any other prophages in *V. cyclitrophicus* (Fig. 3). This implies that there could be a large pool of diverse temperate phages infecting *V. cyclitrophicus*, which is also demonstrated by the 22 tailed prophages clustering into five different genera (Fig. 1). As bacterial strains of different *Vibrio* species have been shown to be naturally transformable upon induction with chitin available in the aquatic ecosystem, loss could be mediated through homologous recombination with strains lacking the prophage [9, 83, 84]. Most prophage genotypes appeared not successful enough to spread widely across *V. cyclitrophicus*. However, an exception was a tailless prophage genotype that might be currently expanding (Fig. 3). We found evidence that this tailless prophage horizontally transferred into three evolutionary separated branches in *V. cyclitrophicus.* In two of these branches, the prophage genotype was also vertically inherited.

### Prophages integrate by site-specific recombination

Having shown that tailless and filamentous prophages were rapidly turned over in *V. cyclitrophicus*, we were interested in how their integration affects the host’s genome architecture. Prophages were specifically recombining into their host’s genome demonstrated by the low number (n = 9) of integration sites, each of which was mostly containing prophages from a single viral realm. For tailed and most tailless prophages, this specificity was mediated by prophage-encoded integrases that were closely related among prophages integrating into the same site (Fig. S8a–b). Furthermore, tailed prophage integrases were diverse, explaining the higher number of integration sites compared to tailless prophages. All filamentous and a few tailless phages recombined site-specifically at the *dif* sites of chromosome 1 and 2. The *dif* sites, conserved sequence motives at each of the two chromosomal termini in *Vibrio* involved in chromosome partitioning [20], have been observed to harbor diverse filamentous prophages in *Vibrio* before [20, 66]. Filamentous phages use host recombinases for integration [20, 85], making the possession of an integrase gene obsolete. In contrast, we observed that tailless prophages targeting the *dif* sites regularly carried integrase genes. Therefore, it remains inconclusive whether tailless prophages recombined into these two hotspots by using host recombinases as suspected before [19], or dependent on their own integrases.

Filamentous and tailless prophages appeared to rescue the function of genomic locations disrupted during integration. Tailless prophages occupying hotspot H1 integrated into the 5′ end of the *dusA* gene coding for a tRNA dihydrouridine synthase, a known target of integrases [86]. The disrupted *dusA* gene was complemented by a prophage-encoded sequence, likely preserving its function. Furthermore, we observed that several filamentous and tailless prophages integrated at the *dif* sites encoded a small sequence motif showing similarity to their host’s *dif* sites possibly ensuring correct chromosome partitioning.

**Figure 4:**
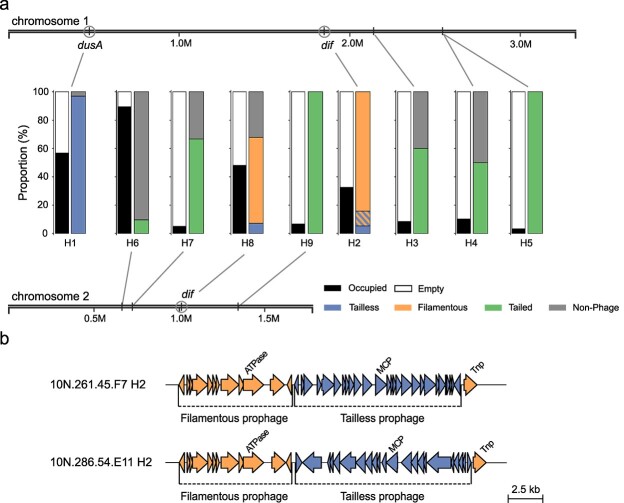
Prophage integration sites in *V. Cyclitrophicus* a) depicted are the locations of prophage integration hotspots H1-H9 on both chromosomes of the prophage-free *V. Cyclitrophicus* 10 N.286.51.B1. Bar plots show the total fill of each integration site in percent (left bar) and which elements the fill is composed of (right bar). Integration sites of tailless and filamentous temperate phages are indicated by circles. The plot shows data of 95 prophages excluding plasmid-encoded prophages and those with inconclusive integration sites. b) the box shows tailless prophages (blue) integrated between the transposase and the rest of a filamentous prophage (orange) in the shared hotspot H2 of *V. Cyclitrophicus* strains 10 N.261.45.F7 and 10 N.286.54.E11. The signature genes for both phages, the double jelly-roll major capsid protein (MCP), and the pI-like ATPase, are indicated. The scale bar shows the sequence length in kb.

Integration sites targeted by filamentous and tailless phages tended to have higher occupancy than those of tailed phages (Fig. 4a). Integration sites containing filamentous or tailless prophages were filled in 33–57%. In contrast, hotspots of tailed prophages were occupied in less than 11% of the 58 strains. One exception was hotspot H6 that was filled in most *V. cyclitrophicus* strains containing a diverse set of elements, such as tailed prophages, transposable elements and defense islands. Our data suggest that tailed temperate phages are more likely to encounter free integration sites, whereas filamentous and tailless temperate phages encounter filled sites more frequently, which might influence the success of prophage integration. Remarkably, filamentous and tailless prophages occasionally co-occurred in a single integration site. Across the *Vibrionaceae*, we found at least 30 such cases. Oftentimes prophages integrated next to each other, however, in *V. cyclitrophicus* we also observed tailless phages integrating into a filamentous prophage separating its transposase from the rest of the filamentous phage genome (Fig. 4b). It remains unclear if these two co-occurring prophages can still produce viable virions. Unlike several related filamentous prophages, both prophages remained inactive upon MMC treatment. This co-integration of tailless and filamentous phages suggests possible competition between viruses of different viral realms, e.g. leading to disruption or inactivation of the resident phage.

### Tailless and filamentous phage genomes possess an accessory gene hot spot

**Figure 5:**
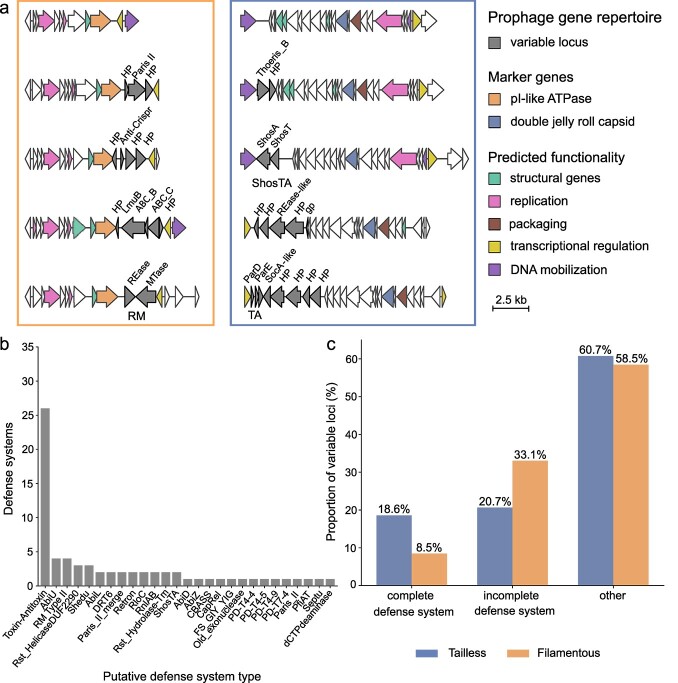
Accessory gene hotspot in filamentous and tailless prophages in the *Vibrionaceae*. a) Example prophage genomes for filamentous (left box) and tailless prophages (right box). Synteny and predicted gene functions are depicted by colored arrows. Proteins within the variable locus are depicted in dark gray with predicted gene functions indicated. DNA mobilization summarizes integrases, excisionases and transposases. White arrows indicate genes, which could not be assigned to a specific function. HP = hypothetical protein, Rease = restriction endonuclease, Mtase =  methyltransferase, LmuB = Lamassu_B, ABC = three component system, gp = glycoprotein, RM = restriction modification system, TA = toxin-antitoxin system. Filamentous prophage hosts (top to bottom): 10 N.286.55.A12, 10 N.239.311.B11, 10 N.261.51.C9, 10 N.237.312.B10, 10 N.239.312.B09. Tailless prophage hosts (top to bottom): 10 N.239.312.A01, 10 N.286.55.E8, 10 N.222.49.B10, 10 N.261.45.E12, 1F169. The scale bar shows the sequence length in kb. b) Types of predicted complete defense systems and coupled toxin-antitoxin genes c) percentage of variable loci with predicted complete defense systems, incomplete defense systems, or other (no defensive genes identified) in filamentous and tailless prophages.

As many prophages carry genes not needed for the viral life cycle [5, 87, 88], we investigated the extent to which prophages with small genomes encode for such accessory genes. Assuming that accessory genes are more frequently exchanged than genes required for infection and reproduction, we searched for variable genes in closely related prophages. To this end, we grouped tailless and filamentous prophages predicted across the *Vibrionaceae* into fine-grained clusters using a protein similarity-based approach (methods). Within clusters, we observed highly conserved synteny between prophage genomes. Single proteins varied in their amino acid sequence presumably caused by gene conversion between prophages. The more striking observation was, however, one locus with variable gene content interrupting this conserved gene order (Fig. 5a). In fact, the variable genes were rarely shared with any other identified prophages. We grouped similar prophages and identified variable loci in 49.4% of unique filamentous and 76.7% of unique tailless prophages (methods). For the remaining prophages, we were lacking enough representatives or variability between genomes to differentiate core and flexible genes, or flanking core genes were absent. The variable loci were often located in proximity to the prophage borders. Flanking core genes were different between the clusters, although one flanking gene frequently annotated as a transcriptional regulator (Table S5). Despite their small genomes, the variable locus comprised up to 11 genes in a single tailless and up to six genes in a single filamentous prophage suggesting selection for these genes.

To understand whether the host might benefit from the variable locus, we investigated the functional gene annotation observing an enrichment for functions related to phage defense. The variable loci we identified in tailless and filamentous prophages across the *Vibrionacaeae* were annotated using databases for both viral and bacterial gene function, complemented with Defensefinder, a tool identifying regions resembling known phage defense systems [45], and a search against the anti-prokaryotic immune systems database [44] (methods). Although most genes in the variable locus remained without functional annotation, 18.6% and 8.5% of the variable loci in tailless and filamentous phage genomes encoded for 27 different putatively complete defense systems (Fig. 5b–c). We found a variety of systems associated with abortive infection, such as toxin-antitoxin systems, retrons or Paris, as well as systems degrading nucleic acids, such as restriction modification systems. Most commonly, we observed toxin-antitoxin systems including those where toxin and antitoxin genes were encoded side by side regardless of not being identified as phage defense systems by Defensefinder. Additional to loci encoding for entire defense systems, 20.7% of tailless and 33.1% of filamentous prophages were predicted to contain incomplete defense systems. We found single defense-associated genes that were flanked by genes encoding for putative effector genes that might complete the defense system. For instance, we found a gene predicted as a Thoeris_B sensor next to a hypothetical protein in a tailless prophage, which together could form a defensive sensor-effector pair (Fig. 5a). We also observed several accessory genes predicted as counter-defenses targeting CRISPR-Cas and the Thoeris defense system. Our data suggest that prophage-encoded variable loci are under selection and possibly defend both host and its prophage from infecting viruses.

## Discussion

Previous studies reported filamentous and tailless prophages across different bacterial taxa suggesting that they are more widespread than previously thought [16–19]. In comparison to tailed prophages, their detection remains challenging also due to their underrepresentation in current databases [15, 19]. By combining chemical induction and comparative genomics for initial prophage candidate prediction instead of using a database-dependent approach, we show that the majority of prophages in naturally co-occurring *Vibrio* are either filamentous or tailless.

Prophage prediction is thought to improve by using high-quality genomes [89]. We could confirm higher prophage recovery, however, our comparison of prophage prediction in high-quality and more fragmented genomes revealed that the detection of filamentous prophages is negatively impacted in particular. Possibly caused by several highly similar prophages within the same genome, assembly breaks might lead to filamentous prophage fragments too small to be detected. In addition, bioinformatic workflows introducing prophage detection size thresholds potentially contribute to the underestimation of filamentous and tailless prophages, which are often as small as defective tailed prophages or phage-inducible chromosomal islands [77, 90, 91].

Filamentous and tailless prophages might balance the negative impact on their host’s fitness by recombining site-specifically into a small number of integration sites. The limitation to few integration hot spots could result from counter selection against the disruption of relevant host-encoded genes, as suggested for tailed prophages before [8]. In fact, tailless and filamentous prophages seemed to restore the function of genomic regions they integrated into. Although this has been described for filamentous phages targeting the *dif* sites [20] and for mobile genetic elements targeting the *dusA* gene [86], we demonstrate here that tailless phages pursue a similar strategy.

Despite their small genome size, filamentous and tailless prophages frequently carried accessory genes encoded in a defined locus, which were often associated with phage defense. Accessory gene hotspots of tailed prophages have recently been shown to contain phage defense systems [5, 6]. We observed defense systems and a few counter-defenses encoded on prophages of different viral realms suggesting that phage defense and counter-defense is widespread. Therefore, less-studied tailless and filamentous prophages might harbor many novel defense and counter-defense systems. In addition, accessory genes might confer nucleic acid immunity that provides protection against other mobile genetic elements apart from newly invading phages [92]. Prophages might also directly benefit from such accessory genes: We identified numerous toxin-antitoxin systems, that could, similar to the previously investigated toxin-antitoxin system PfiAT [93], improve their replication efficiency. The high variation of accessory genes could result from homologous recombination between closely related co-infecting viruses encoding identical flanking regions of the variable locus [25]. Their high variability and possible role in mobile genetic element competition could indicate that accessory genes within the variable locus are under negative-frequency dependent selection. Together, this rapid exchange of the variable loci renders prophages to function as vehicles for phage defense or other virulence genes contributing to the diversification of closely related bacteria.

The variability between prophages of closely related strains indicates the well-known rapid dynamics of prophage gain and loss [9, 10]. However, whether prophages can successfully lysogenize bacteria phylogenetically distant from their previous host, influences how far their genetic information can travel within bacterial communities. Prophages we identified from naturally co-occurring bacteria were mainly shared within bacterial species boundaries, suggesting that both prophage inheritance and phage-mediated horizontal transfers are often species-specific. This supports previous research stating that most phage-mediated gene transfers take place between closely related bacteria and is in accordance with experimental data demonstrating relatively narrow host ranges of temperate phages [94–96]. Recent reports of microbial genetic elements being shared between more distantly related bacteria are challenging this classical view [95]. Yet, this genetic barrier might be special to phages because they require specific receptors to bind their hosts [92].

Although this study is limited to prophages in the family *Vibrionaceae*, previous research suggests that the impact of tailless and filamentous prophages also extends to bacterial communities outside of the *Vibrionaceae*. In particular, tailless and filamentous prophages have been reported in various environments and bacterial taxa [16–18]. Moreover, the integration sites we identified for tailless and filamentous phages are conserved in a wide range of bacteria [86, 97], possibly enabling related prophages to integrate into other bacterial hosts. In conclusion, we here provide a large dataset of 1158 prophages including tailless and filamentous prophages, that we propose to be systematically underestimated in prophage diversity studies. We further show that filamentous, tailless, and tailed prophages are evenly distributed and might play an equally important role in driving the diversification of closely related bacteria beyond the *Vibrionaceae*.

## Supplementary Material

2024-09-25_Supplement_Prophages_wrae202

Table_S1_wrae202

Table_S2_wrae202

Table_S3_wrae202

Table_S4_wrae202

Table_S5_wrae202

## Data Availability

Reference host reads and genomes were deposited with the accession numbers indicated in Table S2. Genomes of the 107 well-curated prophages are available under the GenBank accession numbers PQ189284–PQ189390 (Table S2). Reads for MMC sequenced cultures were submitted to the SRA under Bioproject number PRJNA1137887 (Table S2).
