# Supplementary material for: Tailless and filamentous prophages are predominant in marine Vibrio
Source: ISME J. 2024 Oct 18;18(1):wrae202. doi: 10.1093/ismejo/wrae202 (PMC11630473; doi:10.1093/ismejo/wrae202)
Supplement: 2024-09-25_Supplement_Prophages_wrae202 [file 2024-09-25_supplement_prophages_wrae202.docx]

Tailless and filamentous prophages are predominant in marine *Vibrio*.

Kerrin Steensen^1,2^, Joana Séneca^3,4^, Nina Bartlau^1^, Xiaoqian A. Yu^1^, Fatima A. Hussain^5^, Martin F. Polz^1^

# Supplementary Methods

### High quality reference genomes of *Vibrio cyclitrophicus*

Considering that closed genomes are beneficial for prophage discovery, we sequenced 58 selected bacterial isolates using Oxford Nanopore technology (ONT) (Table S2). Bacteria were streaked on 1.5 % agar plates and incubated overnight. Single colonies were transferred into 7 ml liquid 2216MB and grown in a rotator at medium speed ON. Cells were pelleted at 12,000 x g and 4°C until the supernatant cleared (10-40 min).

DNA was extracted from bacterial pellets using the Power Soil Pro Kit (Qiagen) and sequenced in two rounds (“N01” and “N02”). In Brief, the DNA was prepared for sequencing using the rapid barcoding sequencing kit (SQK-RBK110.96, Oxford Nanopore Technologies) following the manufacturer’s protocol. Barcoded samples were sequenced in R9.4.1 flowcells on a MinION Mk1b (N01, FLO-MIN106D, Oxford Nanopore Technologies) and on a Promethion P24 (N02, FLO-PRO002, Oxford Nanopore Technologies). The DNA sequencing was carried out using Minknow (v. 22.03.6, Oxford Nanopore Technologies). Raw ONT reads were basecalled using Guppy (v. 6.1.1) in super accuracy mode and assembled using flye (v. 2.9-b1768, (1)) with “–nano-hq”. The Illumina reads for polishing that were obtained as described in “*Vibrionaceae* genome collection” were trimmed using cutadapt (v. 3.1, (2)). The assembly was polished three times using short read Illumina data with minimap2 (v. 2.17, (3)) and racon (v. 1.4.3, (4)) and two times using long-reads with medaka (v. 1.6.1, github.com/nanoporetech/medaka). Reads were mapped to the assemblies using minimap2 (v. 2.17, (3)), read mappings were converted using samtools (v. 1.12, (5)) and read coverage was calculated using metabat2 (v. 2.15, (6)). If the polished assemblies contained contigs smaller than 500 bp, they were excluded from this study.

We resequenced genomes not closed after the first runs (“N03”): High molecular weight DNA was extracted with the Monarch HMW DNA extraction kit for tissue (New England Biolabs) with the protocol for Gram negative bacteria. Extracts were left at 4°C for at least one week before quantification and fragment estimation. Samples were diluted and equimolarly barcoded using the SQK-RBK114.96 (Oxford Nanopore Technologies) with the following modifications: the sample input was increased to >80ng/sample, added 0.2ul barcode/sample, and added 0.5ul of rapid adaptor to the barcoded library. About 20 fmol of a >60 kb library was loaded on a R10.4.1 flowcell (FLO-PRO114, Oxford Nanopore Technologies) and sequenced for 48 h on a Promethion P2 solo (Oxford Nanopore Technologies) using Minknow (v. 23.11.4, Oxford Nanopore Technologies). Flowcell light shields were used. Reads were basecalled using either Dorado (v. 7.8.2) or Guppy (v. 6.1.1) using super accuracy mode (Table S2). Raw reads shorter than 500bp and with average Q-scores <15 were removed using chopper (v0.6, (7)) and assembled using flye (v. 2.9.1, (1)) with “–nano-hq”. The assembly was polished once with medaka (v. 1.11.1, github.com/nanoporetech/medaka) using model “r1041_e82_400bps_sup_g615”. Contigs smaller than 1kb were excluded from the polished assemblies. Genomes were manually inspected and depending on the overall coverage of the main contigs, lower coverage contigs (either <10x or <30x) were removed. The genome quality, completeness and contamination values were checked using QUAST (v. 5.0.2, (8)) and CheckM (v. 1.1.1, (9)).


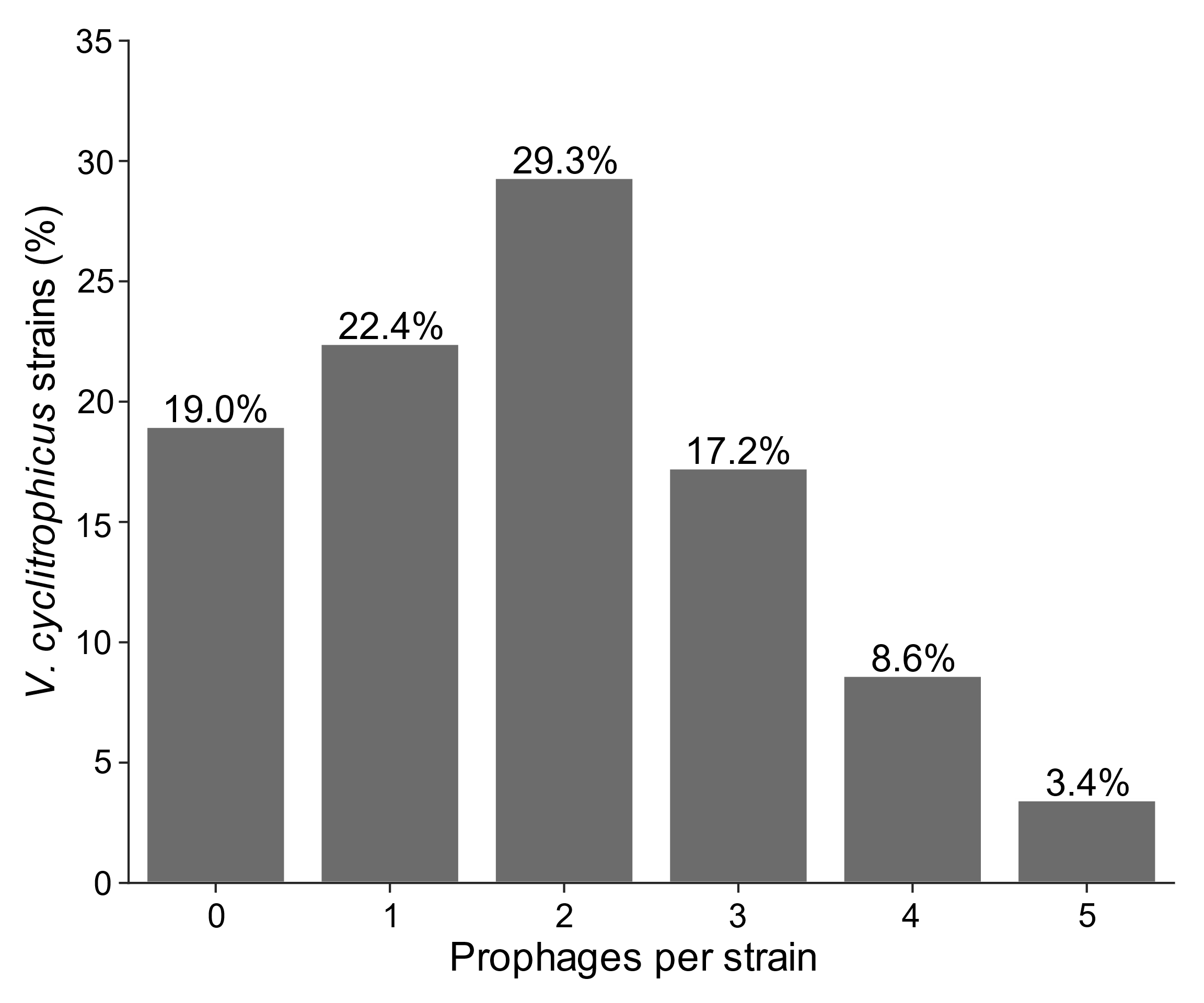


Figure S1. Prophage distribution in *Vibrio cyclitrophicus*

Histogram showing the distribution of prophages carried per bacterial strain in 58 *Vibrio cyclitrophicus* genomes. Strains carried 1.8 prophages on average.


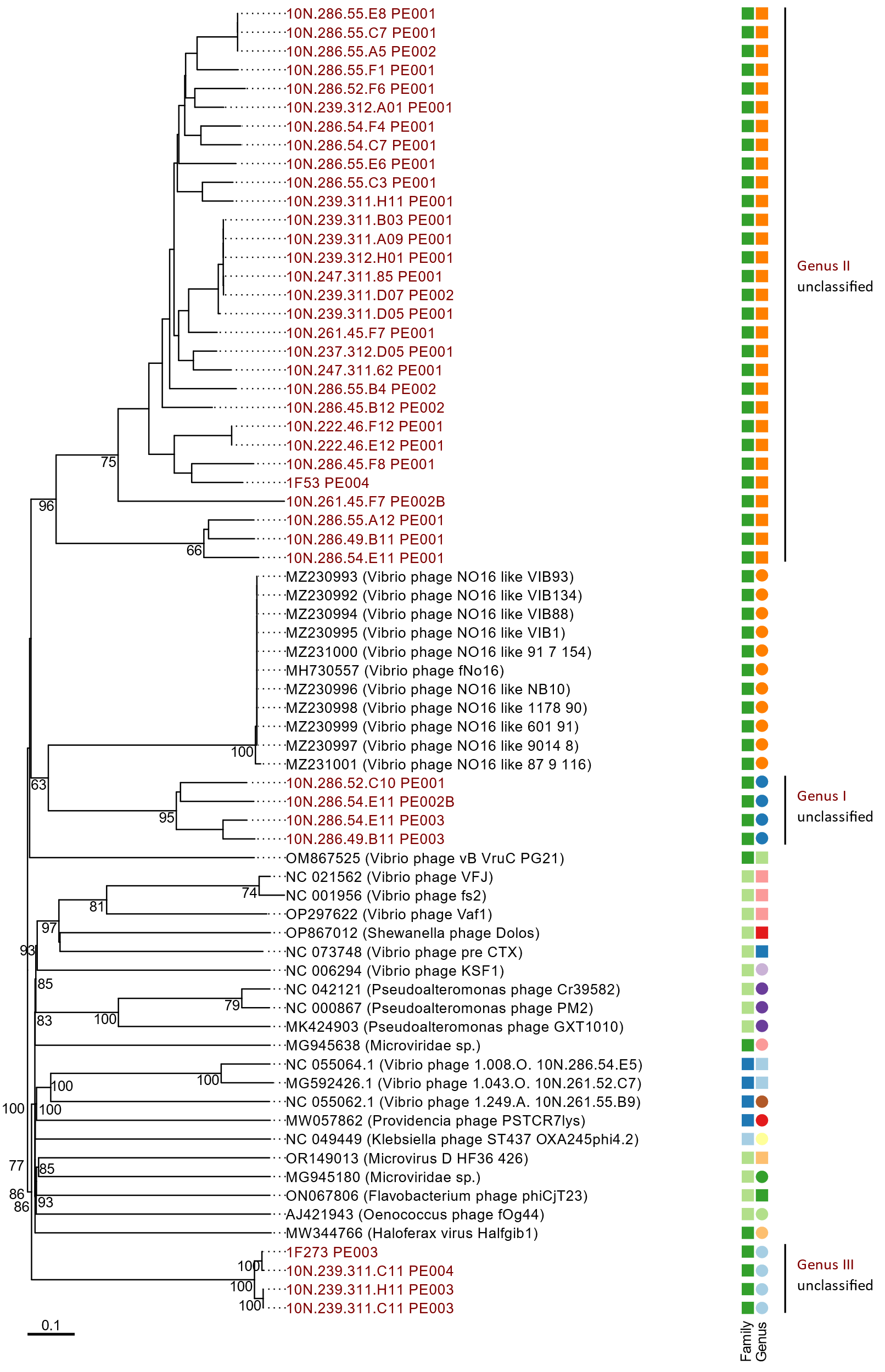


Figure S2a. Classification of tailless prophages.

Balanced minimum evolution tree inferred from intergenomic distances between genome sequences of identified (a) tailless (b) filamentous and (c) tailed prophages (red) and selected known tailless phages. Pairwise distances were calculated using the Genome-BLAST Distance Phylogeny formula D0. Values at the branches denote the support from 100 pseudo-bootstraps. Taxonomic classification was predicted using VICTOR and is represented by colored symbols and the number of the respective genus indicated. The scale bar represents the number of nucleotide substitutions per site.


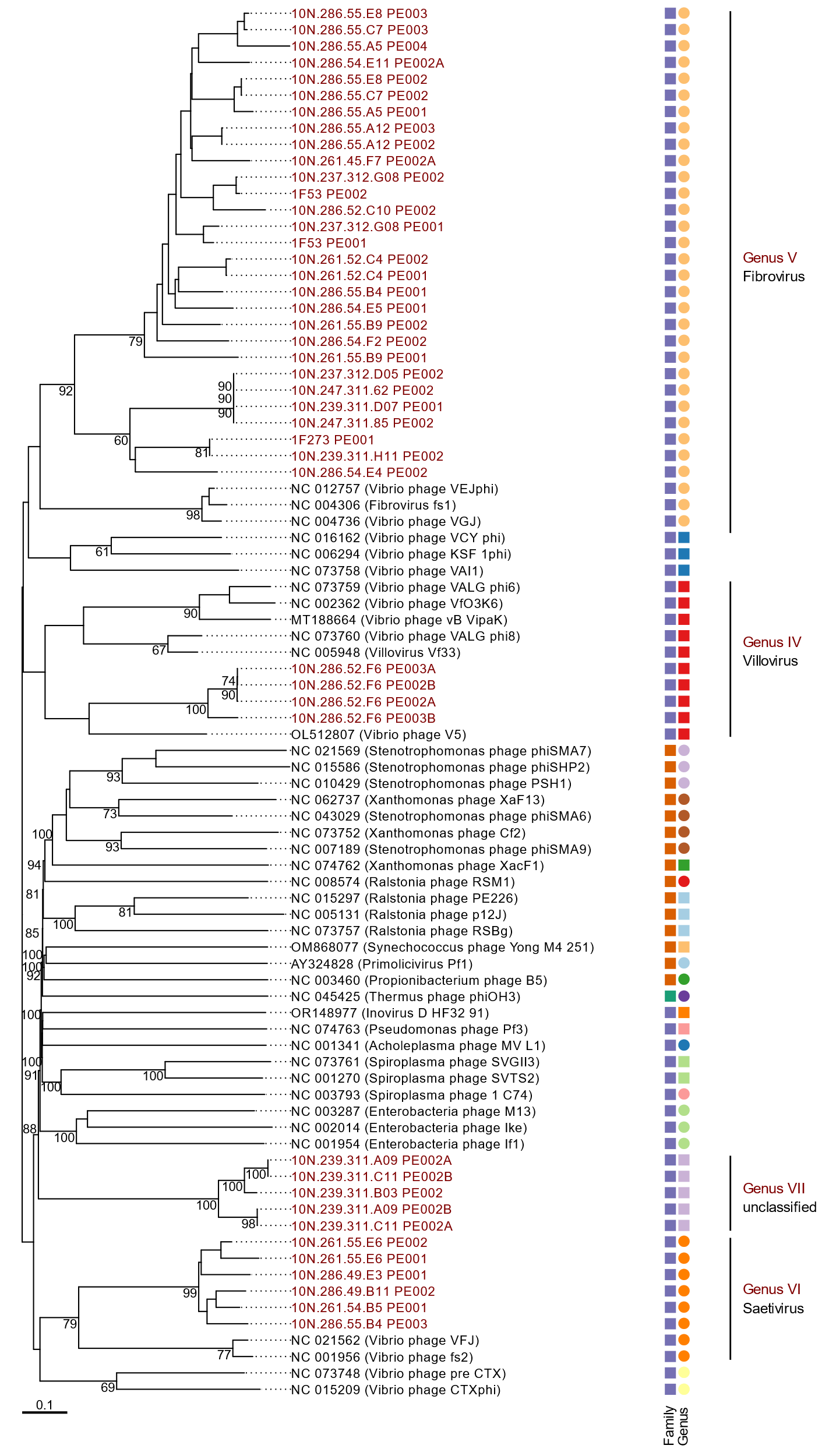


Figure S2b – Classification of filamentous prophages.


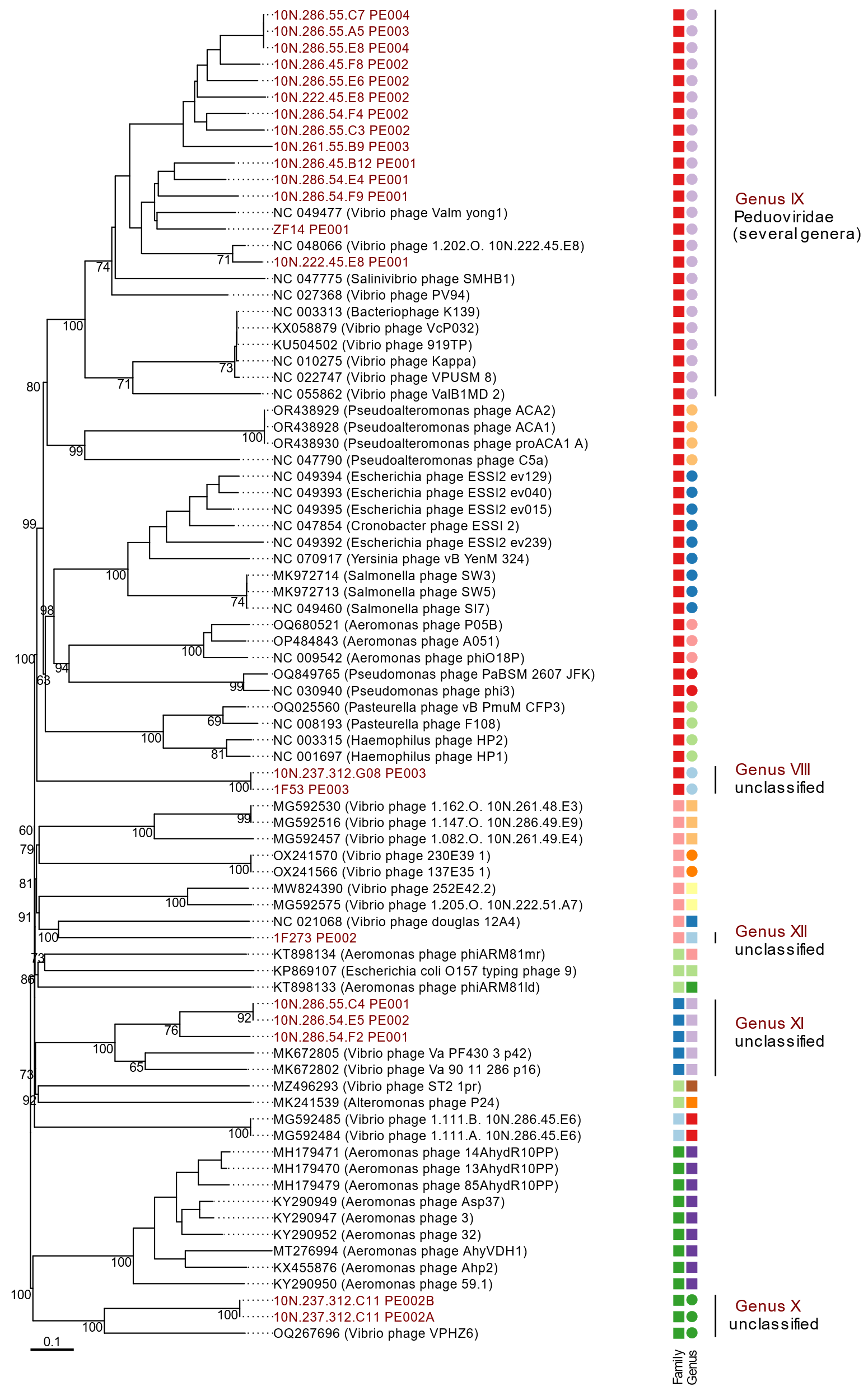


Figure S2c – Classification of tailed prophages.


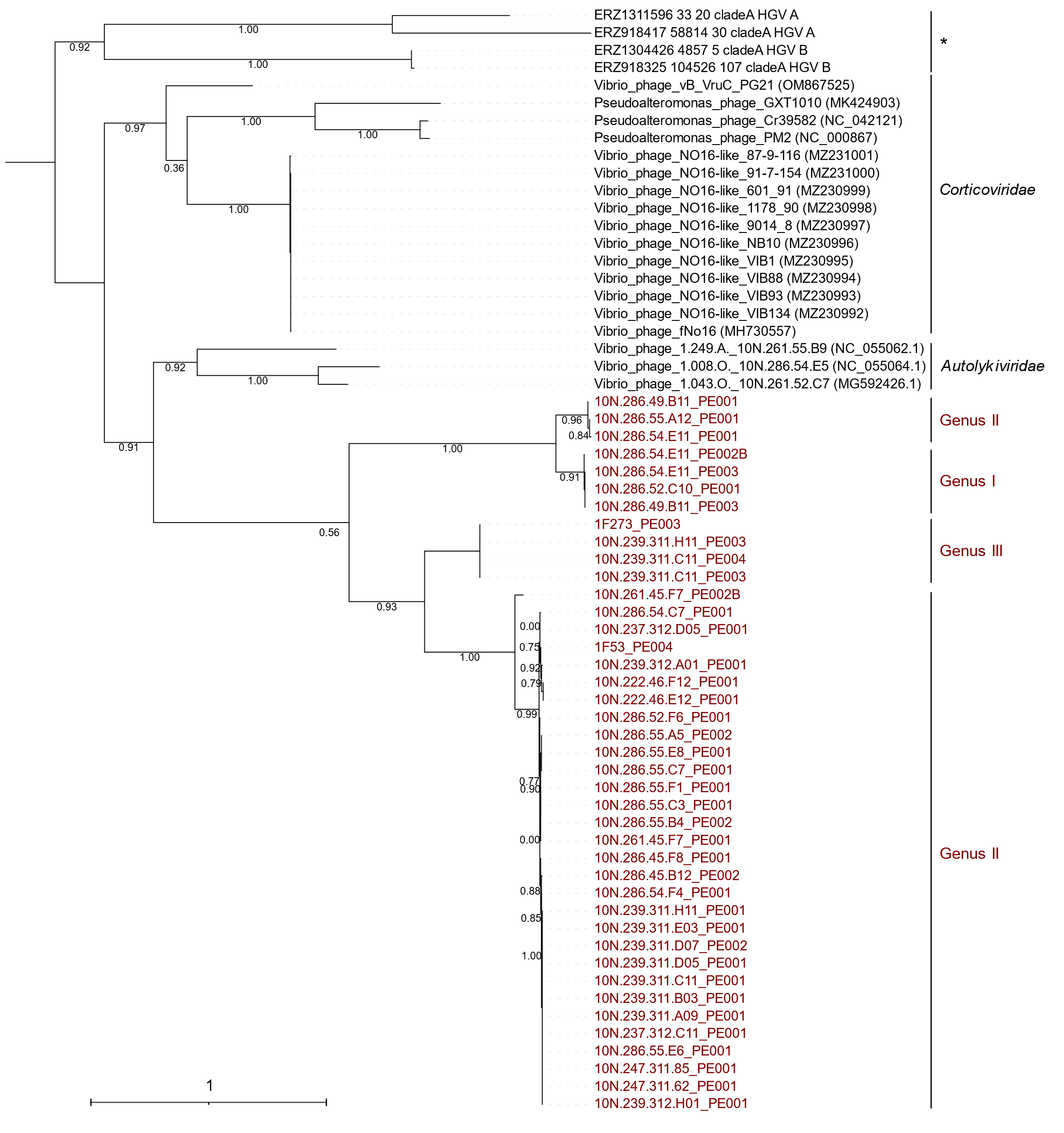


Figure S3a - Marker gene comparison to known tailless phages
Phylogenetic tree based on the alignment of double jelly-roll major capsid proteins of identified prophages and known representatives of the *Vinavirales and Autolykiviridae*. Prophages identified in this study are highlighted in red. Additional double jelly-roll major capsid proteins marked with an asterisk originate from major capsid proteins identified human gut genomes (“clade A”) in a previous study (Yutin et al., 2022). The scale bar represents amino acid substitutions per site. Bootstrap values are given in a range from 0 to 1.


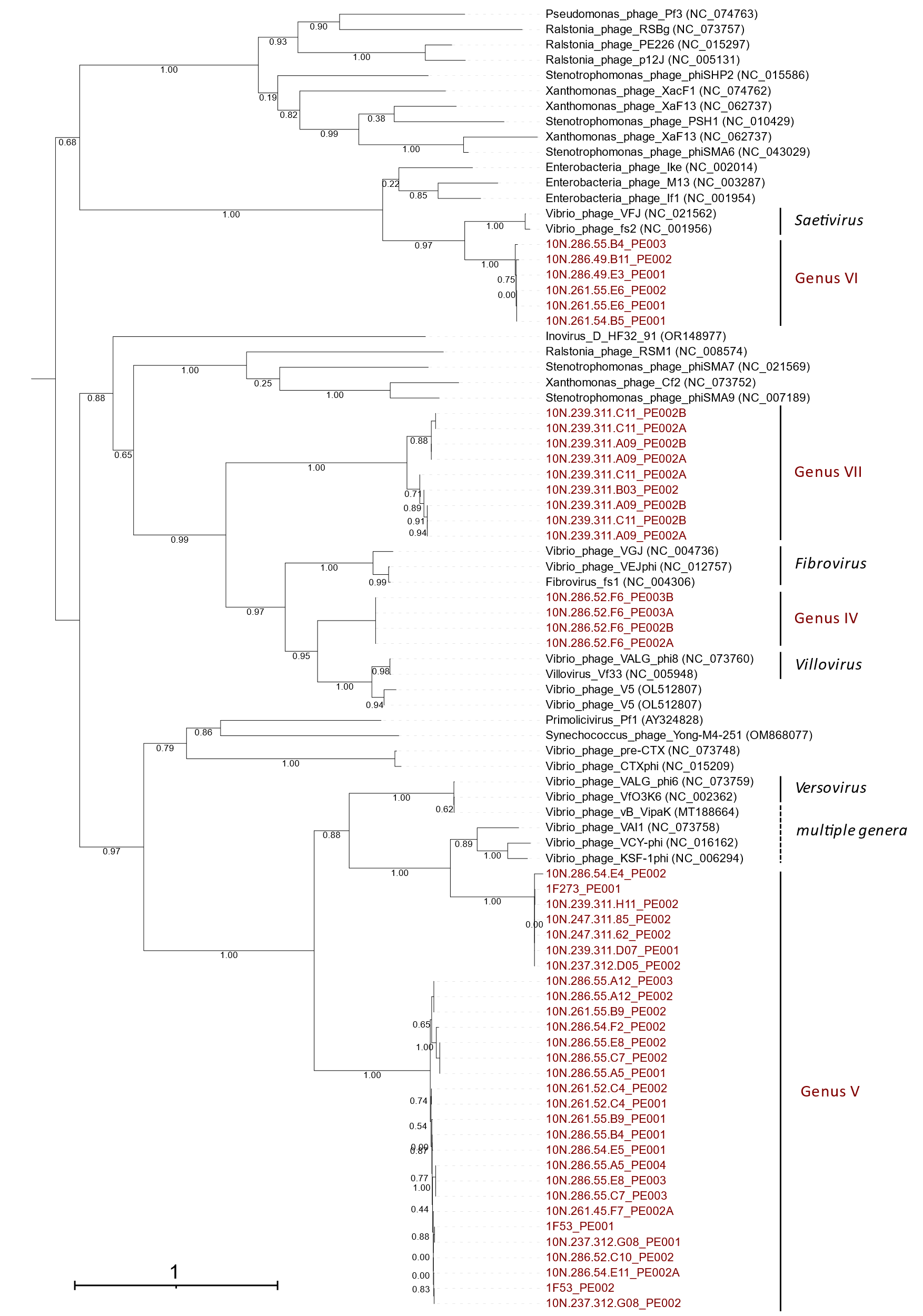


Figure S3b - Marker gene comparison to known filamentous phages
Phylogenetic tree based on the alignment of the pI-like ATPase genes of identified prophages and known representatives of the *Inoviridae*. If two copies of pI-like ATPases are present, both are shown. Prophages identified in this study are highlighted in red. Known filamentous phages represent the majority of genera currently present in the ICTV taxonomy. The scale bar represents amino acid substitutions per site. Bootstrap values are given in a range from 0 to 1.


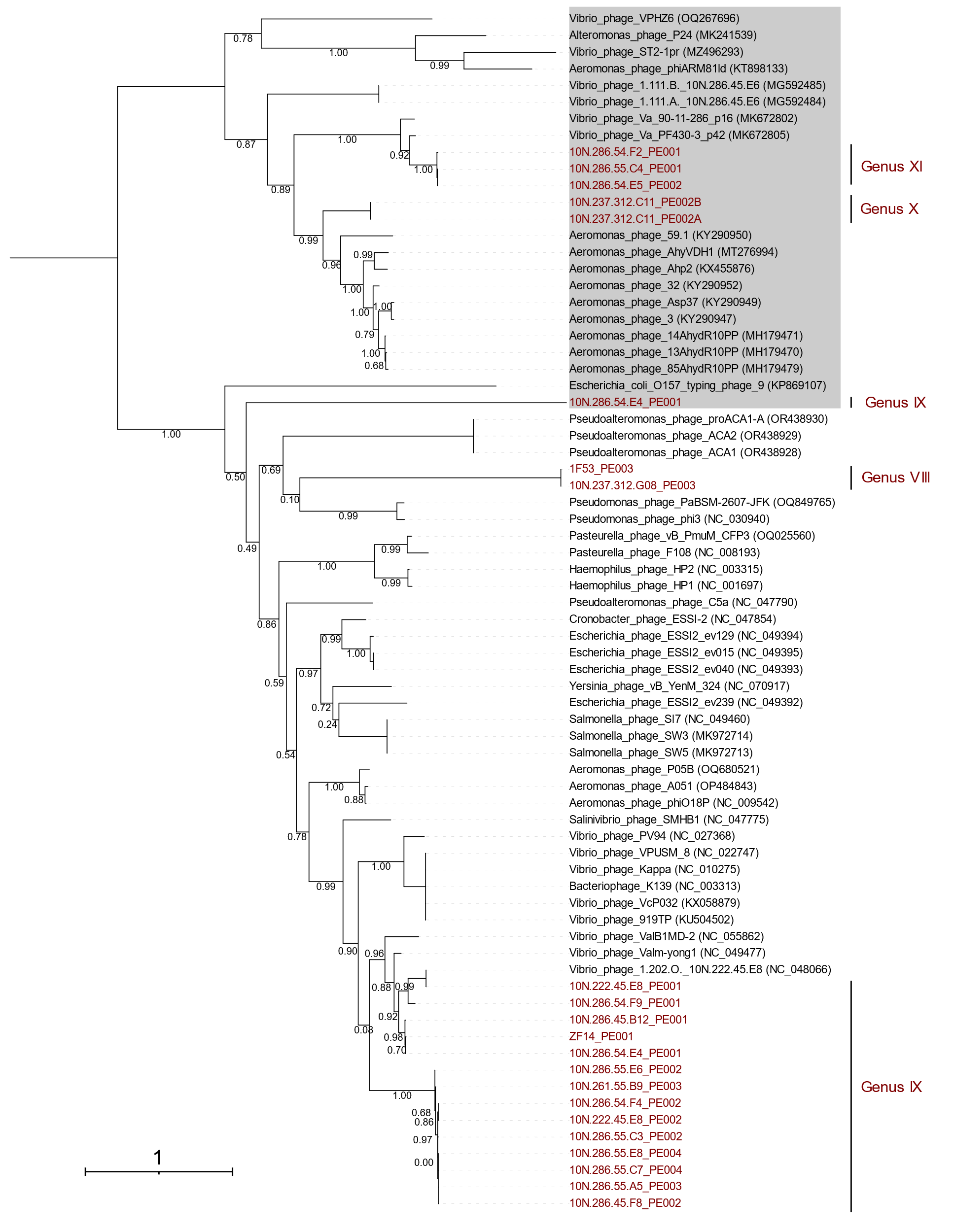


Figure S3c - Marker gene comparison to known tailed phages
Phylogenetic tree based on the alignment of the large subunit (gray box) or endonuclease subunit of terminase genes of identified prophages and known representatives of the *Caudoviricetes*. If two terminase copies are present, both are shown. Prophages identified in this study are highlighted in red. Tailed phages without a clear terminase prediction are excluded. The scale bar represents amino acid substitutions per site. Bootstrap values are given in a range from 0 to 1.


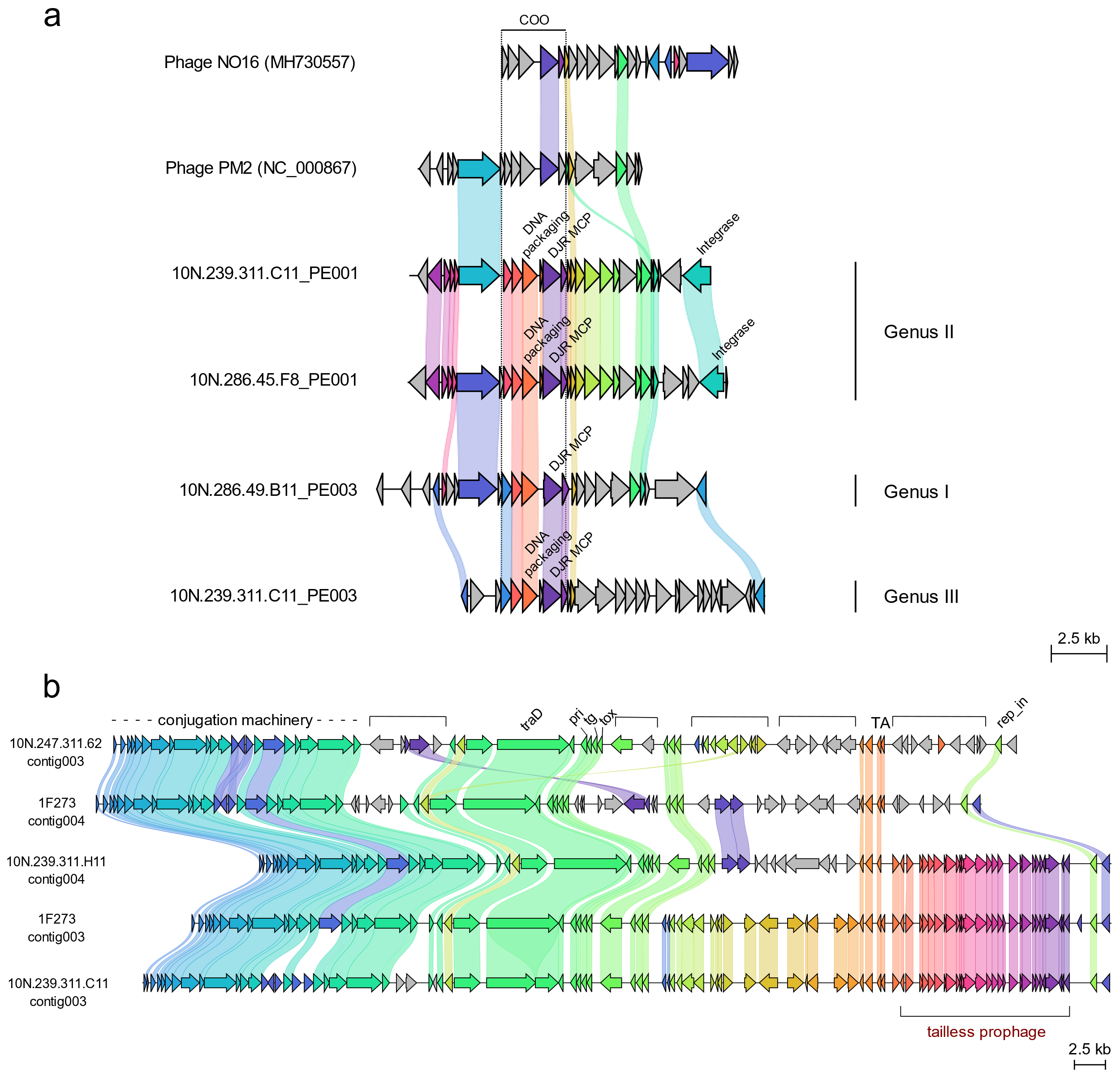


Figure S4 – Genomic maps of tailless phage genomes and plasmids.

a) Representative genomes of tailless phage genera I-III are shown in comparison to known tailless phages PM2 and NO16. Gene annotations for the double jelly-roll major capsid protein (DJR MCP), the DNA packaging ATPase and Integrase genes were added if applicable. The gene order of the identified phages from Genera I-II partially resembled the ones of PM2 and NO16, for example in the marked region with conserved gene order (CGO). b) Plasmids carrying tailless prophages of genus III and plasmids with a similar backbone identified in V. cyclitrophicus. Core gene annotations denote transfer gene D (traD), a primase (pri), transglycosylase (tg), a toxin (tox), a toxin-antitoxin pair (TA) and a replication initiation protein (rep_in). Flexible regions are marked by brackets. In both plots, genes are depicted as arrows, and genes with amino acid identity above 30% are colored and connected. The scale bar shows the sequence length in kb.


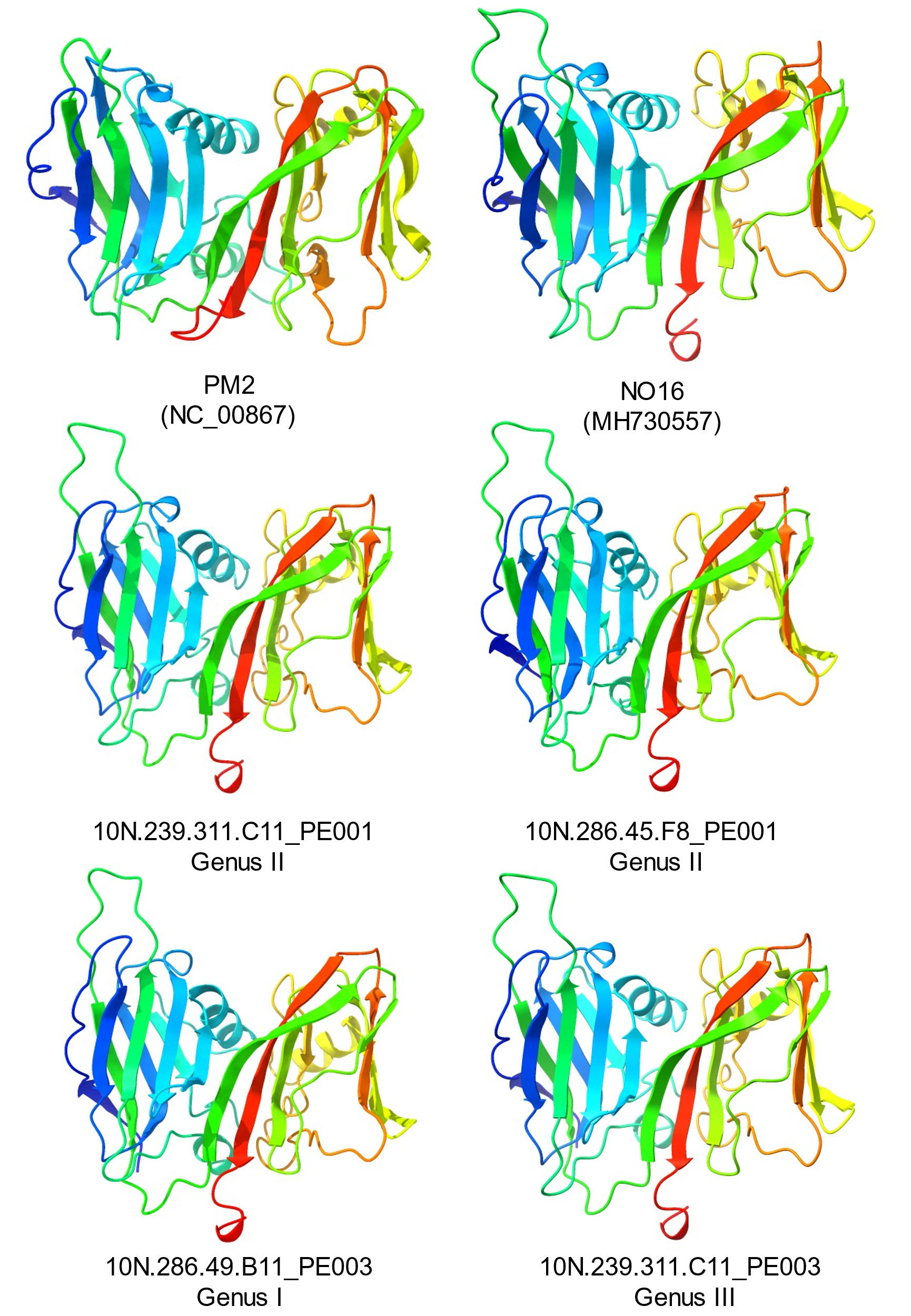


Figure S5. Structural conservation of double jelly-roll major capsid proteins encoded by tailless phages.

Monomers of the crystal structure of the major capsid protein of phage PM2, and structural predictions of the monomers of major capsid proteins obtained by alphafold for phage NO16 and four representative tailless phages from genera I-III.


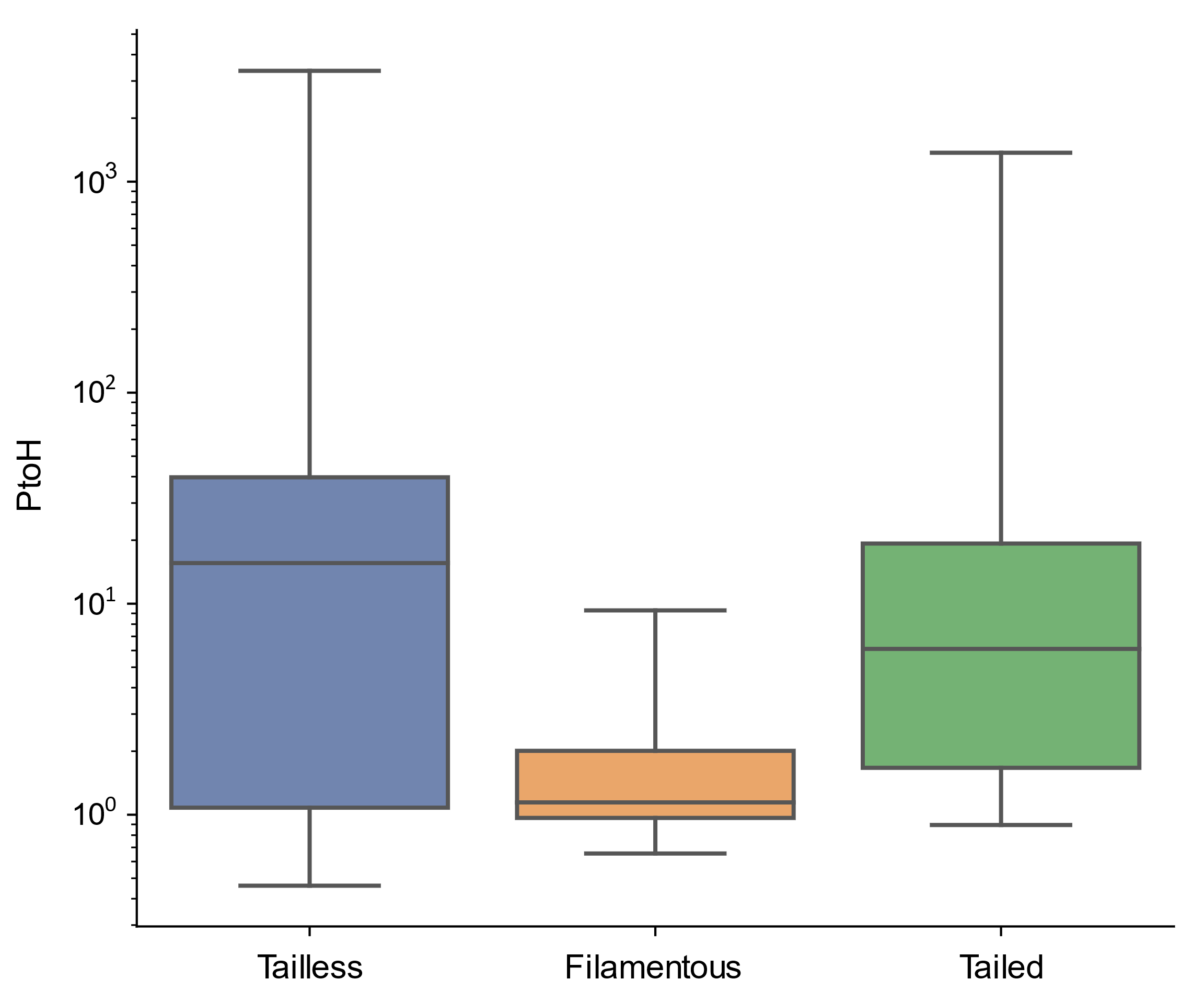


Figure S6. Prophage activity

Boxplots show the relative coverage of prophages compared to their genomic neighborhood given with the phage-to-host ratio (PtoH) as a measure of the prophage activity. Each boxplot represents the phage-to-host ratios of prophages per viral realm on a logarithmic scale. Whiskers show the range between the minimum and maximum values, and box edges represent the lower and upper quartile of the phage-to-host ratios. The median is given by a horizontal line.


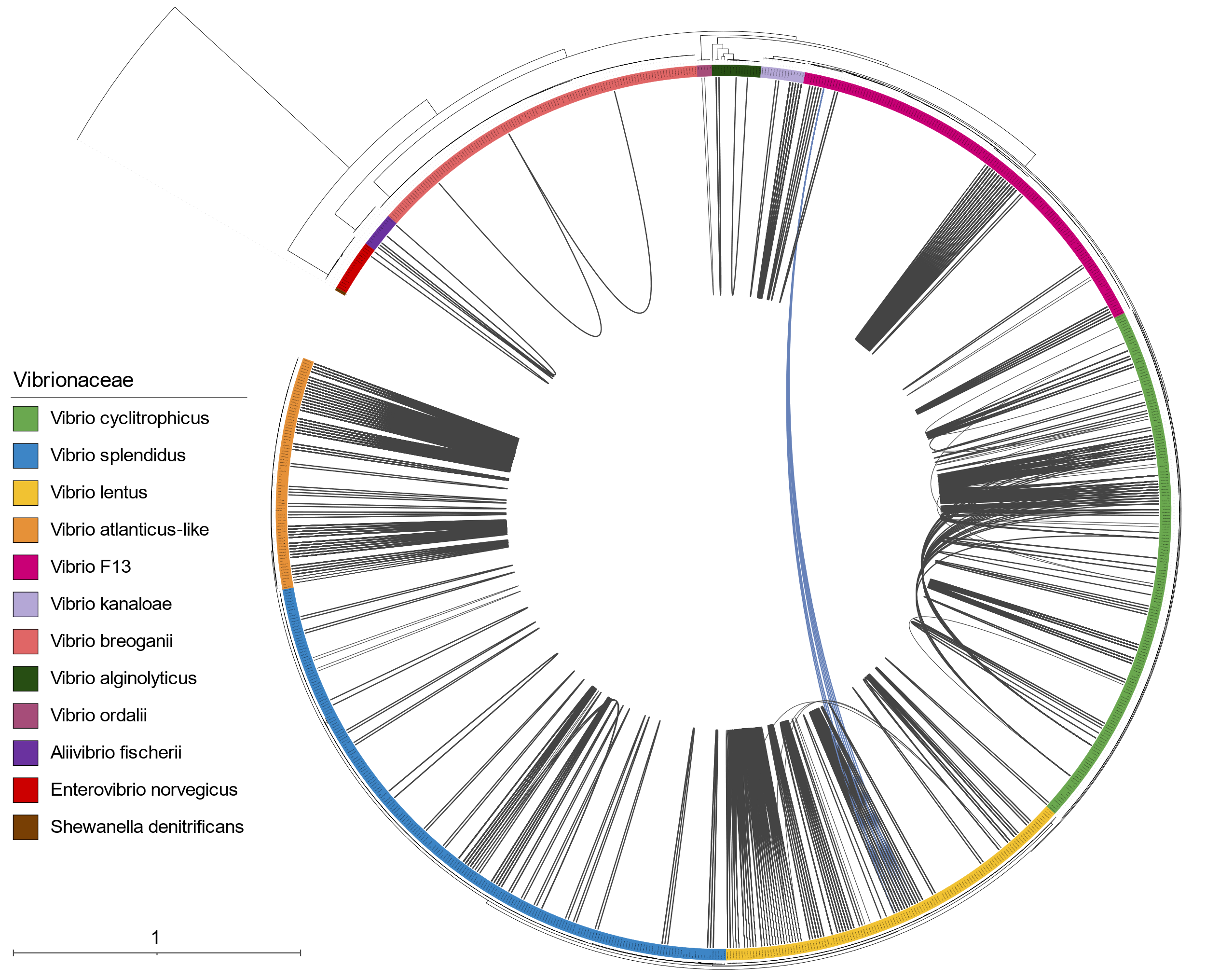


Figure S7 - Shared prophages across the *Vibrionaceae*

Shared prophages (ANI >99.9%, full coverage) across co-occurring populations of different marine *Vibrionaceae* (colored by species). Phylogenetic relationships of the different *Vibrionaceae* strains are based on single copy ribosomal protein. The scale bar denotes substitutions per site. Transfers within population are depicted in black. Transfers between populations are highlighted according to the viral realms (blue = tailless prophages).


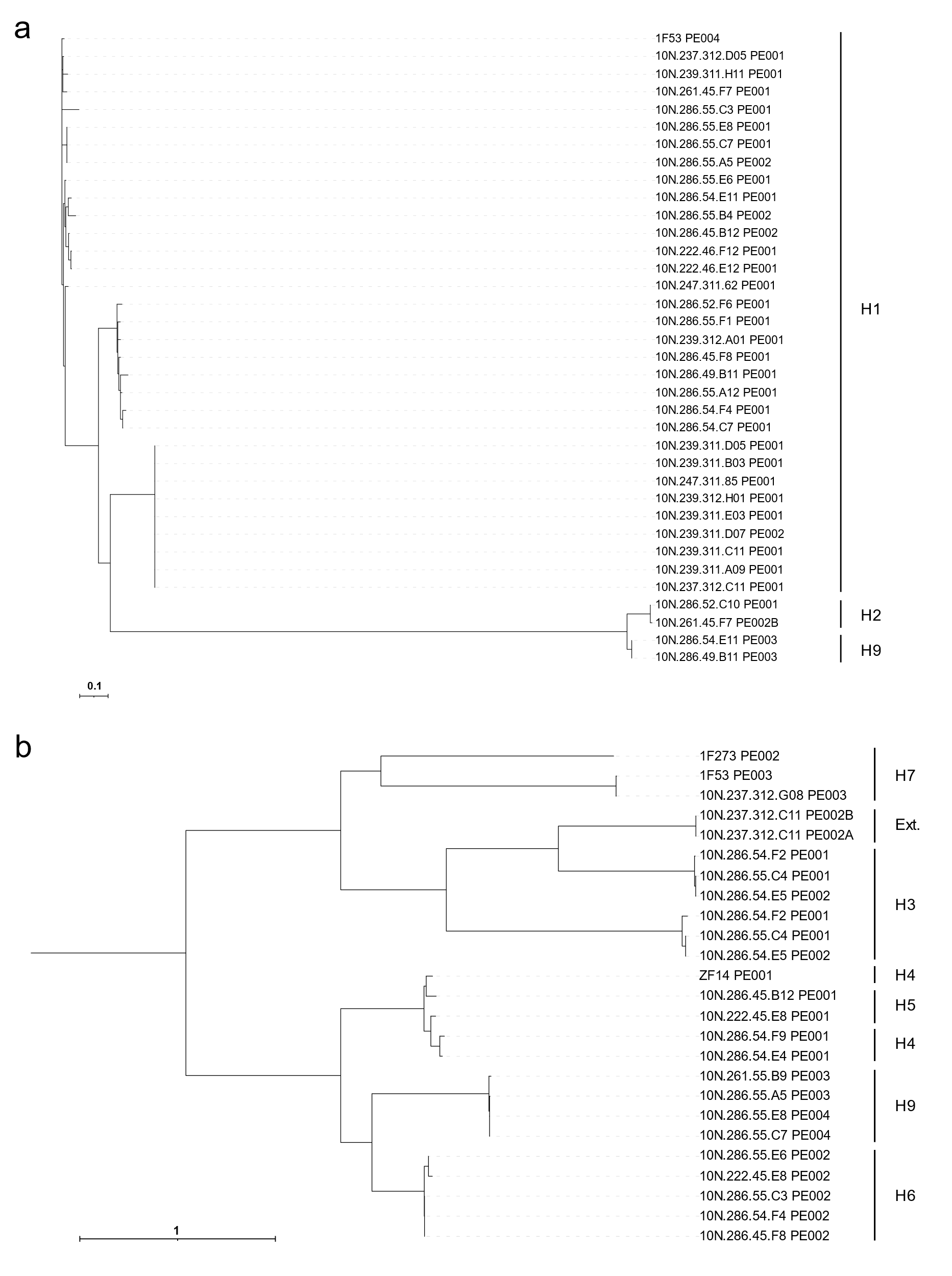


Figure S8 – Prophage integrases and associated integration sites.

Phylogenetic trees based on protein alignments of integrase genes in tailless (a) and tailed (b) prophages. The tree scale represents substitutions per site. Phylogenetically related integrases are mostly carried by prophages integrating into the same genomic location, indicated by hotspots H1-H9.

# References

1. Kolmogorov M, Yuan J, Lin Y, Pevzner PA. Assembly of long, error-prone reads using repeat graphs. *Nat Biotechnol*. 2019; 37(5):540–6.

2. Martin M. Cutadapt removes adapter sequences from high-throughput sequencing reads. *EMBnet J*. 2011; 17(1):10–2.

3. Li H. Minimap2: pairwise alignment for nucleotide sequences. *Bioinformatics*. 2018; 34(18):3094–100.

4. Vaser R, Sović I, Nagarajan N, Šikić M. Fast and accurate de novo genome assembly from long uncorrected reads. *Genome Res*. 2017; 27(5):737–46.

5. Danecek P, Bonfield JK, Liddle J, Marshall J, Ohan V, Pollard MO, et al. Twelve years of SAMtools and BCFtools. *GigaScience*. 2021 Jan 29; 10(2):giab008.

6. Kang DD, Li F, Kirton E, Thomas A, Egan R, An H, et al. MetaBAT 2: an adaptive binning algorithm for robust and efficient genome reconstruction from metagenome assemblies. *PeerJ*. 2019; 7:e7359.

7. De Coster W, Rademakers R. NanoPack2: population-scale evaluation of long-read sequencing data. *Bioinformatics*. 2023 May 4; 39(5):btad311.

8. Gurevich A, Saveliev V, Vyahhi N, Tesler G. QUAST: quality assessment tool for genome assemblies. *Bioinformatics*. 2013; 29(8):1072–5.

9. Parks DH, Imelfort M, Skennerton CT, Hugenholtz P, Tyson GW. CheckM: assessing the quality of microbial genomes recovered from isolates, single cells, and metagenomes. *Genome Res*. 2015; 25(7):1043–55.
